# Improved tRNA prediction in the American house dust mite reveals widespread occurrence of extremely short minimal tRNAs in acariform mites

**DOI:** 10.1186/1471-2164-10-598

**Published:** 2009-12-11

**Authors:** Pavel B Klimov, Barry M OConnor

**Affiliations:** 1 University of Michigan, Museum of Zoology, 1109 Geddes Ave, Ann Arbor, Michigan 48109-1079 USA

## Abstract

**Background:**

Atypical tRNAs are functional minimal tRNAs, lacking either the D- or T-arm. They are significantly shorter than typical cloverleaf tRNAs. Widespread occurrence of atypical tRNAs was first demonstrated for secernentean nematodes and later in various arachnids. Evidence started to accumulate that tRNAs of certain acariform mites are even shorter than the minimal tRNAs of nematodes, raising the possibility that tRNAs lacking both D- and T-arms might exist in these organisms. The presence of cloverleaf tRNAs in acariform mites, particularly in the house dust mite genus *Dermatophagoides*, is still disputed.

**Results:**

Mitochondrial tRNAs of *Dermatophagoides farinae *are minimal, atypical tRNAs lacking either the T- or D-arm. The size (49-62, 54.4 ± 2.86 nt) is significantly (p = 0.019) smaller than in *Caenorhabditis elegans *(53-63, 56.3 ± 2.30 nt), a model minimal tRNA taxon. The shortest tRNA (49 nt) in *Dermatophagoides *is approaching the length of the shortest known tRNAs (45-49 nt) described in other acariform mites. The D-arm is absent in these tRNAs, and the inferred T-stem is small (2-3 bp) and thermodynamically unstable, suggesting that it may not exist in reality. The discriminator nucleotide is probably not encoded and is added postranscriptionally in many *Dermatophagoides *tRNAs.

**Conclusions:**

Mitochondrial tRNAs of acariform mites are largely atypical, non-cloverleaf tRNAs. Among them, the shortest known tRNAs with no D-arm and a short and unstable T-arm can be inferred. While our study confirmed seven tRNAs in *Dermatophagoides *by limited EST data, further experimental evidence is needed to demonstrate extremely small and unusual tRNAs in acariform mites.

## Background

Atypical, non-cloverleaf tRNAs were first demonstrated computationally and experientially for secernentean nematodes [1-4]. Despite the loss of either the D- or T- arm, these tRNAs preserve the L-shaped tertiary conformation of the normal tRNA, with the exception that the interstem angle slightly increases [5]. Apparently atypical tRNAs retain normal function, thus exemplifying the concept of a minimal tRNA [2,6], a structure approaching the minimal level of simplicity necessary for a functional translational system. Minimal tRNAs were later found in vinegaroons, buthid scorpions, spiders and acariform mites [7-15], although in *Dermatophagoides *and some other taxa, cloverleaf tRNAs were also inferred. The distribution of minimal tRNAs across distantly related taxa is attributed to multiple independent evolutionary events [7]. In secernentean nematodes, non-cloverleaf tRNAs were believed associated with the presence of minimal rRNA [1], however, a recent study links modifications of tRNAs and the enzyme EF-Tu delivering aminoacyl-tRNAs to the ribosome [16,17].

Two major tRNA detection programs, tRNAscan-SE [18] and ARWEN [19], implemented the 'bizarre' tRNAs [1] of nematodes, however, the performance of these programs is not expected to be perfect when applied to mites. In the first acariform taxon where tRNAs were characterized (*Leptotrombidium pallidum*), most of them are even shorter than atypical tRNAs of nematodes [9]. This is also true for *Dermatophagoides*, where several tRNAs could not be confidently recovered by the two programs and probably represent even more 'bizarre' structures as compared to secernentean nematodes. On the other hand, short and unstable structures or cloverleaf structures were also inferred previously in *Dermatophagoides*, suggesting the need of their independent verification. In another acariform mite, *Steganacarus magnus*, only 8 tRNAs could be detected [11]. Another potential difficulty with the inference of tRNA genes may involve mismatches in their acceptor stems that can be edited and corrected after transcription [7,20,21]. With these mismatches, tRNA genes may not be different from a random DNA sequence, and, therefore, may be difficult or impossible to infer. Posttranscriptional editing of the tRNA acceptor stem was hypothesized for spiders having highly destabilized and divergent acceptor stems in contrast to well paired and evolutionary conserved D- and anticodon arms [7,22,23]. Mismatches in the acceptor stem do occur in acariform mites, including *Dermatophagoides*, although to a much lesser extent, indicating that a similar mechanism may exist in these organisms.

A few examples given above suffice to show that inference of mite mitochondrial tRNAs may be extremely difficult and error prone, especially when comparative data are absent. Therefore, we verified previously published annotation of *Dermatophagoides pteronyssinus *[12], the only other member of Astigmata, then-studied for tRNA structure, with our data on *D. farinae *and a few other astigmatid taxa. Various lines of evidence were used: (i) comparative sequence conservation, including compensatory mutations; (ii) RNAscan-SE and ARWEN analyses; (iii) similarity with GenBank data (to account for atypical tRNAs, only anticodon arm sequences were used in BLAST searches); (iv) polyadenylation sites (EST data), which may indicate the 5' end of a tRNA; (v) thermodynamic stability, especially when alternative structures where inferred. In the analyses below we call four unusually short tRNAs "non-canonical" because they can not be confidently inferred by available tRNA search programs.

## Results and Discussion

### Mitochondrial genome of *Dermatophagoides farinae*

**Figure 1:**
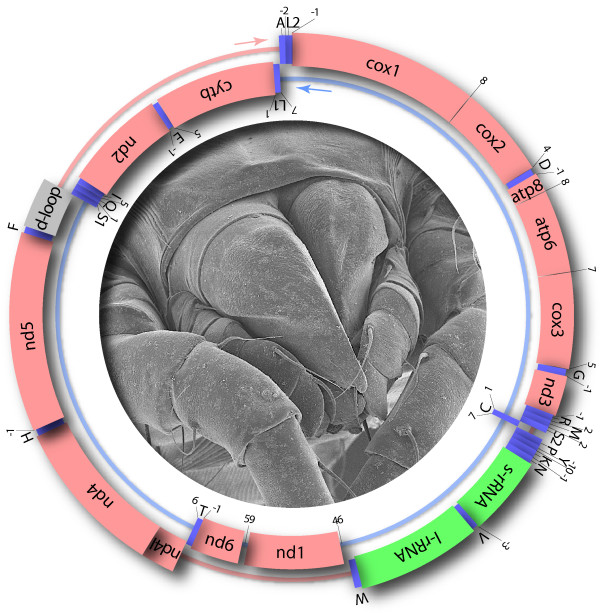
**Mitochondrial genome of *Dermatophagoides farinae***. Distances (nt) between genes regions (coding sequences and structural RNA) are indicated by numbers; overlaps are indicated by negative numbers. For transport RNAs, single-letter abbreviations are used.

**Figure 2:**
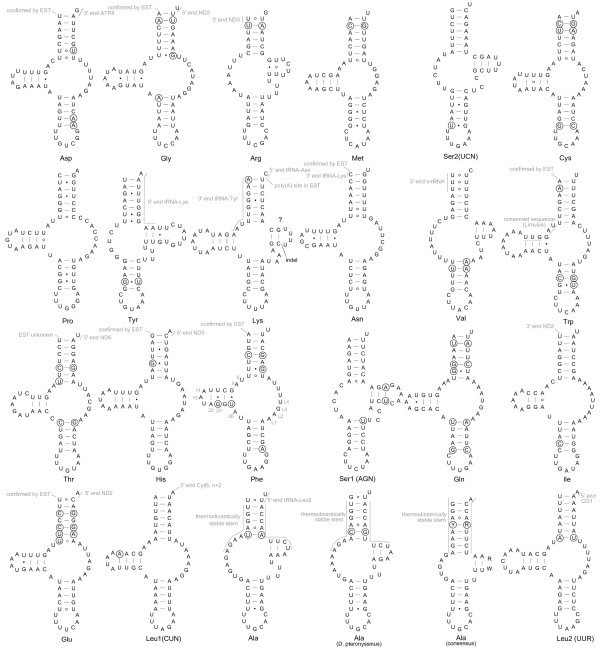
**Transfer tRNAs of *Dermatophagoides farinae***. Compensatory mutations are indicated by circles. If the 3' end of the individual mRNA (as found by a polyadenylated tail in ESTs) is immediately contiguous to a tRNA gene [27], then the 5' end of this tRNA is indicated by the "confirmed by EST". ESTs are not available for all regions. Residues forming tertiary interactions in tRNA-Phe are annotated after [3]. tRNA-Ala of *D*. *pteronyssinus *and the consensus between *D. farinae *and *D*. *pteronyssinus *are also given.

Similarly to most bilaterians, the mitochondrial (mt) genome of *Dermatophagoides farinae *is a covalently closed circular DNA (14,266 nt) encoding 13 polypeptides, 2 ribosomal RNAs, and 22 tRNAs on two strands. All translation products are members of the multi-subunit complexes that couple oxidative phosphorylation [24], whereas the ribosomal RNAs and tRNAs are part of mitochondrial protein biosynthesis machinery [25]. The majority strand forms 2 mt-rRNAs (*l-rRNA *and *s-rRNA*), 15 mt-tRNAs and encodes 9 polypeptides (nucleotide frequencies: T 0.447, C 0.110, A 0.267, G 0.176; GC-skew 0.2314, AT-skew -0.2531) (Additional file 1). The minority strand forms 7 tRNAs and encodes 4 polypeptides (Fig. 1). However, unlike many other bilaterians, the majority strand is the light strand (as defined by molecular weight). Available EST data suggest that the mitochondrial genes are transcribed as polycistronic (multigenic) transcripts, which are cleaved and polyadenylated to yield mature mRNAs [26]. Processing of the majority of polycistronic units is probably governed by the precursor mt-tRNA structures as found elsewhere [27]. Most of tRNAs are very short and do not have either the T- or D-arm (Fig. 2; Table 1). Similar short tRNAs were found in vinegaroons, spiders, scorpions, and other acariform mites, but typical cloverleaf tRNAs were also recovered in these taxa [7-15]. Polyadenylation sites in the mRNA mark the 5' ends of a tRNA, suggesting that precursor tRNAs are cleaved precisely at their 5' end (see below). We hypothesize that the discriminator nucleotide at the 3' end is not encoded and added posttranscriptionally in many tRNAs (see below). The small and large subunit rRNA show many features common for minimal rDNA previously only known for two model organisms, the nematode *Caenorhabditis elegans *and the trypanosome *Leishmania tarentolae *[28-30]. The most striking similarity shared by these organisms is the absence of several large helices in the GTPase region in the large subunit rRNA (our data, unpublished). The most frequent codon on the majority strands is TTT (Phe), and on the minority strand is ATA (Met), highlighting asymmetric strand bias. There is a clear preference for GT-rich codons on the majority strand, while AC-rich codons were more frequent on the minority strand. However, only at the third position were the differences statistically significant (Additional file 2). As indicated by EST data, poly(A) stretches were implicated in the creation of complete stop codons from incomplete T codons [31] in *ND3*, *ND6*, *ND4*, and *ND5*. Alternative polyadenylation is found in the transcript of gene *ND6 *in *D. pteronyssinus *(Additional files 3, 4). The D-loop of *D. farinae *is variable in length in a single individual (heteroplasmy), with two indels (0-34 and 0-4 nt, respectively) occurring inside two major AT-repeats (Additional file 3).

**Table 1:** Transfer tRNAs in *Dermatophagoides farinae *and *D. pteronyssinus*

**An**^**a**^	**Cn**^**b**^	tRNA	**Str**^**c**^	**Len**^**d**^	**Ant**^**e**^	**Str**^**f**^	**TRN**^**g**^	**ARW**^**h**^	**BLAST**^**i**^	**Prev**^**j**^	**Cf**^**k**^	**5' overlap**^**l**^	**3' overlap**^**m**^
1-3	y	Asp/D	+	54	GTC	tv	Y	Y	D		y	-	*ATP8 *(1)
4-7	y	Gly/G	+	57	TCC	tv	Y	Y	G		y	-	*ND3 *(1)
8-14	n	Arg/R	+	49	TCG	d	-	y	R	tv^n^		*ND3 *(1)	-
15-18	y	Met/M	+	52	CAT	tv	y	-	M			-	-
19-25	y	Ser2/S2	+	52	TGA	d	-	y	S2, W	tv^o^		-	-
26-28	y	Cys/C	-	53	GCA	d	Y	Y	C	**A**		-	-
29-32	y	Pro/P	+	57	TGG	tv	Y	y	P			-	-
33-40	n	Tyr/Y	+	55	GTA	d	-	y	Y	**V**^p^		-	K (10)
41-44	y	Lys/K	+	62	TTT	c/tv	y	y	K, W, C, L2	tv^q^		Y(10)	N(1),-
45-47	y	Asn/N	+	55	GTT	tv	Y	Y	N		y	K(1)	-
48-53	n	Val/V^r^	+	52	TAC	d	-	-	V, Y, *l-rRNA*	***l-rRNA***		*s-rRNA*(3,2)	-
54-57	y	Trp/W	+	58	TCA	tv	Y	Y	W, S2, F		y	-	-
58-61	-	-^s^	+	44	'GGT'	-	-	-	-	**Y**		-	-
62-67	y	Thr/T	-	55	TGT	tv	Y	y	T			-	*ND6 *(1)
68-70	y	His/H	+	56	GTG	tv	Y	Y	H		y	-	*ND5 *(1)
71-77	y	Phe/F	+	55	GAA	tv	Y	Y	F, W		y	-	-
78-80	y	Ser1/S1	-	52	TCT	d	-^t^	y	S1, S2	d^u^		-, Q (2)^v^	-
81-83	y	Gln/Q	-	54	TTG	tv	Y	Y	Q, W			-, I (2)^v^	-, S1(2)^v^
84-88	y	Ile/I	-	53	GAT	tv	y	-	I			-	-, Q (2)^v^
89-92	y	Glu/E	-	54	TTC	tv	y	Y	E, W	tv^w^	y	-	*ND2 *(1)
93-95	y	Leu1/L1	-	55	TAG	tv	Y	Y	L1	tv^x^		-	-
96-100	n	Ala/A^y^	+	50	TGC	d	-	y	-^z^	**C**^aa^		-	-
101-103	y	Leu2/L2	+	57	TAA	tv	Y	Y	L2			-	*COX1*(1)

^a ^refers to IDs from the detailed analyses (Additional file 5)

^b ^Canonical, can be found confidently either in tRNAscan-SE or ARWEN

^c ^Strand

^d ^Length (*D. farinae *only)

^e ^Anticodon

^f ^tRNA secondary structure: tv = TV-replacement loop tRNA, d = D-replacement loop tRNA, c = cloverleaf tRNA

^g ^tRNAscan-SE analysis: Y = high score or unambiguous tRNA; y = low score or ambiguous tRNA; - = failed to find any tRNAs

^h ^ARWEN analysis: designations as for tRNAscan-SE

^i ^BLAST similarity search of tRNA anticodon helix restricted to mitochondrial genomes of arthropods

^j ^Previously inferred for *D. pteronyssinus*, if either gene region (bold) or its secondary structure is different from our reconstruction

^k ^The 5' end of a tRNA is confirmed by EST data (a polyadenylated site immediately upstream of the tRNA 5' end). For others tRNAs these data are not available. Approximated if EST data are missing in one species.

^l ^in all cases overlaps with the 3' end of a gene. The overlap length (nt) is given in parentheses. tRNAs genes are given using single letter designations. If different in the two species, given as two values separated by a comma, for *D. farinae *and *D. pteronyssinus*, respectively.

^m ^In all cases overlaps with 5' end of a gene; format as in previous column

^n ^17 nt overlap with ND3 3' end

^o ^12 nt overlap with 3' end tRNA-Met

^p ^5' end overlaps with 5' of tRNA-Lys by 7 nt, 3' end overlaps with 3' end of tRNA-Pro by 11 nt

^q ^3' part is different from our structure

^r ^putative; alternatively part of *l-rRNA *start where it forms stable secondary structure with 6-nt "anticodon", variable loop, and 5-nt stem.

^s ^not a tRNA, non-coding region between tRNA-Trp and *ND1*

^t ^also tRNA-Ser2

^u ^3' acceptor and T-stems are separated by 1 nt. These two stems are not separated in our structure (as in the typical tRNA)

^v ^*D. pteronyssinus *only

^w ^5' acceptor stem and D-stem (3 bp) are separated by 1 nt. These two stems are separated by 2 nt (as in the typical tRNA); D-stem is 4 bp-long in our structure; and the acceptor stem is situated at n-1 position in our structure

^x ^3-bp D-stem (4-bp in our structure)

^y ^alternatively non-coding region between tRNA L1-tRNA L1-L2 structure

^z ^one hit on *Steganacarus magnus *may represent tRNA-Ala (originally designated as non-translated intergenic spacer).

^aa ^cloverleaf, 3' end overlaps with tRNA-Leu1 by 5 nt.

### tRNA-Asp

*Dermatophagoides *retains the presumed ancestral chelicerate gene order "Asp-*ATP8*-*ATP6*-*COX3*-Gly", and this TV-loop tRNA is part of it. In *D. farinae*, the acceptor stem has 2 mismatches (Fig. 2), while *D. pteronyssinus *has only one mismatch. Two putative compensatory mutations were detected in the anticodon stem (Fig. 2). The 5' end of this structure is confirmed by EST data for *D. pteronyssinus *[GenBank: EX162413MW DP0389]. For *D. pteronyssinus *only, BLAST search returned a positive alignment with the anticodon arm of a collembolan (Additional file 5, 1). It is interesting that in the same position of the oribatid mite, *Steganacarus magnus*, ARWEN suggests tRNA-Asp (as a D-loop) (Additional file 5, 3), and BLAST search aligns it with the same collembolan mentioned above. However, this tRNA has 2 mismatches in the anticodon stem and is thermodynamically unstable. We refrain from inferring this structure in *S. magnus*.

### tRNA-Gly

The position of tRNA-Gly represents the presumed ancestral chelicerate gene arrangements (see above for tRNA-Asp), and this tRNA (TV-loop) was inferred with relatively high support by tRNAscan-SE and ARWEN. No significant structural differences were detected, except for the 7-bp acceptor stem predicted by the former program, as opposed by the 8-bp stem inferred by ARWEN. EST data [GenBank: CB284188DF0911] provide evidence that this structure may be a functional structure, however, the exact 5' end of it cannot be determined because the polyadenylation site is located inside of a 4A (*D. farinae*) or 3A (*D. pteronyssinus*) homopolymeric region. We present a conservative reconstruction having a 7-bp acceptor stem (Fig. tRNA), which is consistent with the canonical tRNA. The discriminator nucleotide (the 3' dangling end) of this tRNA may be the first position of a possible start codon (TTG) for the gene *ND3*. Two compensatory mutations occurred in the acceptor stem and one such mutation occurred in the anticodon stem. BLAST search of the anticodon helix of this tRNA returned many positive matches, including that of an acariform mite (*Leptotrombidium deliense *[GenBank: NC_007600]). In *S. magnus*, ARWEN suggests tRNA-Gly (cloverleaf) at this position. Similarly to many putative tRNAs of this species, this tRNA has mismatches in the anticodon stem and is thermodynamically unstable (Additional file 5, 7). Nevertheless its anticodon arm sequence returned numerous positive hits for other arthropods, including ticks (Additional file 5, 7), indicating that at least this sequence is evolutionarily conserved and has a biological function.

### tRNA-Arg (non-canonical)

In accordance with tRNAscan-SE, this structure was inferred as a TV-loop for *D. pteronyssinus*, with highly unstable D- and acceptor stems having 1 (out of 2) and 3 (out of 7) unpaired base pairs, respectively [12]. Furthermore, it has a very large overlap with the coding gene *ND3*, thus violating the tRNA punctuation model of RNA processing [27]. The program mfold renders this structure as having a positive minimum free energy (4.00) (Additional file 5, 8), indicating that even without isolated base pairs this structure is unlikely to be formed at the standard temperature. Similarly, the acceptor stem of *D. farinae *is thermodynamically unstable and its position does not correspond to that of *D. pteronyssinus*.

Secondary structures of tRNA, as inferred by ARWEN in our analyses, differ substantially in *D. farinae *and *D. pteronyssinus*. In the former species, ARWEN predicts it has the typical cloverleaf structure with both D- and T- arms, while in the latter species it was a D-loop, lacking the D-arm and with a very reduced T-arm (Additional file 5, 8; Additional file 6, 1). Similarly, if shorter sequences are considered, an array of structures, including TV-loops, were inferred by ARWEN (Additional file 5, 10-12). Although, their acceptor stems are situated at different positions (at least their 5' parts for TV-loops) (Additional file 6, 2-3), t the anticodon stem is the same across all these structures. As indicated by minimum energy analysis, all these structures may be unstable (positive MFE), except for the D-loop structure, which has a negative MFE (Additional file 5, 8-9).

Consensus in terms of thermodynamic stability and positional similarity between the two species is only possible when the ARWEN D-loop structure of *D. pteronyssinus *is used. We give a conservative folding of *D. farinae *(Fig. 2) based on the reconstruction of *D. pteronyssinus *(Additional file 5, 8; Additional file 6, 1). If correct, one mutation in the putative acceptor stem should be considered as compensatory (Fig. 2), and an additional compensatory mutation is present if the acceptor stem is located at the n+1 position. Most of *Dermatophagoides *tRNAs (except for tRNA-Ser1 and -Ser2, which are evolutionarily conserved D-replacement loops, see below) are TV-loops, so our preliminary D-loop structure may be questioned. However, this finding is supported by the following: (i) In TV-loop tRNAs, the variable loop region is purine-rich (which is necessary for maintaining tertiary conformation), while in D-loops it is not [e g., [6]]. Our structure follows the pattern of the canonical D-loop tRNA. A D-loop tRNA-Arg was also suggested for three trombidiform mites (genera *Unionicola, Walchia, Leptotrombidium*), which similarly have a very small 8-9 nt T-arm [10], the arachnid *Mastigoproctus giganteus *[7], and two dipluran species of the genus *Campodea *[32]. (ii) Even though TV-loop tRNAs were found (Additional file 5, 10-12; Additional file 6, 2-3), their 5' parts of the acceptor stem are different, and no stable and homologous D-arm can be inferred across the two species in these reconstructions. Mismatches in the acceptor stem may be assumed (because they can be edited later after transcription [7,20,21,33-35], but mismatches in either D- or T stems are not known to be edited. (iii) Similarly, the cloverleaf structures of the two species disagree in the position of their acceptor stems, with that of *D. farinae *situated downstream from a 6-nt indel, and thus it is unlikely to exist.(iv) All reconstructions, except for D-loops, were unstable, with positive MFEs.

In summary, the above evidence (thermodynamics, BLAST hits, similarities with tRNA-Arg of trombidiform mites) suggest that a D-loop tRNA (Fig. 2) should be preferred here.

### tRNA-Met

Inference of this tRNA is not straightforward because there is no agreement between the two programs. tRNAscan-SE finds a structure only in *D. farinae*, and this a low-score TV-loop tRNA-Met (0.55). The same tRNA was also inferred previously [12] for *D. pteronyssinus*. ARWEN presents an alternative structure with D- and T-arms for both species. These tRNA have a relatively high threshold value (95-100%), but they have many mismatches in the putative acceptor stems and are thermodynamically unstable (MFE>0) (Additional file 5, 17-18). If a sequence corresponding to the tRNAscan-SE structure is entered to ARWEN, then it will reconstruct a similar structure for both *D. farinae *(Fig. 2) and *D. pteronyssinus*. We consider these TV-loop structures, having acceptable thermodynamic parameters (Additional file 5, 16) and one compensatory mutation in the acceptor stem, as most probable for *Dermatophagoides*. The following conserved sequences (as compared to trombidiform mites [10]) were detected: AGCTA, AAGCT (D-arm, 5 and 3' parts, respectively), GGTCATA (5' fragment of the anticodon arm). Anticodon arm sequence of this tRNA returns many positive hits for various arthropods (Additional file 5, 15-16).

### tRNA-Ser2(UCN)

This tRNA cannot be determined unambiguously, although there is some evidence that this is a D-loop. Previous reconstructions for both species [12] have a D-arm and lack a T-arm (TV-loop). Both have a highly destabilized acceptor stem, and *D. farinae *also has a destabilized D-arm and a large overlap with the previous tRNA-Met, indicating that non-anticodon stem parts of this tRNA were probably inferred incorrectly. The program tRNAscan-SE confirms the reconstruction only for *D. pteronyssinus*. In contrast, ARWEN suggests that in the two species, the 5' end of the acceptor stem is 1 nucleotide shorter and shifted at the n-1 position as compared to the previous structure [12]. This shift makes a fully complimentary acceptor stem in both species. MFE for these structures are 0.56 and -2.9, for *D. pteronyssinus *and *D. farinae *(Additional file 5, 19, 20), respectively.

The above inferences were based on the alignment limited to the previous reconstructions [12], which is only supported by tRNAscan-SE for *D. pteronyssinus *(Additional file 5, 19, 20). If flanking regions are also considered, various tRNAs are inferred by ARWEN (Additional file 5, 21-22) with -Ser2 shared among all analyses, while results of tRNAscan-SE are similar to the limited analysis restricted to the Dermauw et al. [12] fragment. tRNA Ser2, as detected by ARWEN, does not have a D-arm but has a T-arm instead (D-loop) (Fig. 2) and does not overlap with the preceding tRNA. It has fully complimentary anticodon and acceptor stems and is much more stable thermodynamically, with MFEs of -5.73 and -2.9 for *D. pteronyssinus *and *D. farinae*, respectively. It is unusual for chelicerate tRNAs (except for tRNA-Ser1) to lack a D-arm [7]. However, similarly to our inference (Fig. 2), the absence of a D-arm in tRNA-Ser2 was reported in the oribatid mite *Steganacarus magnus *[11], species of the prostigmatan mite genera *Leptotrombidium *[8,9], *Unionicola *and *Walchia *[10], a whip scorpion, *Mastigoproctus giganteus *[7], two spider genera, *Ornithoctonus huwena *[36] and *Aphonopelma *[7], annelids [37], and certain nematodes [38,39] and gastropods [40]. We consider the structure inferred by ARWEN (Fig. 2) as the preferred tRNA-Ser2.

It is interesting that for the anticodon arm of this tRNA, BLAST returned many significant alignments with tRNA-Trp (= complement to tRNA-Ser2), and the scores of these alignments were much higher than for tRNA-Ser2 (16 versus 11 matches). In addition, the ancestral chelicerate gene order (tRNA-Trp-tRNA-Cys and *ND1*-tRNA-Ser2) will be favored if the identity of our tRNA-Ser2 and -Trp is switched (see below). Although this scenario cannot be completely ruled out, there is some evidence that, in fact, our reconstruction of tRNA-Ser2 (D-loop) is correct: (i) mutations at the last nt of the 5' part of the anticodon stem are such that they are compensatory only for tRNA-Ser2; (ii) no tRNA-Trp consistent for both species could be found either by tRNAscan-SE or ARWEN (data not shown); high-score tRNA-Trp can be inferred at another location and, in both *Dermatophagoides *and *Steganacarus*, it has a certain evolutionarily conserved pattern specific to tRNA-Trp (see below). Unfortunately, we could not find tRNA-Ser2 (T-stem) specific pattern [10] in either *Dermatophagoides *or *Steganacarus*.

The same problem with the identity of tRNA-Ser2 and -Trp exists in *Steganacarus magnus*: originally it was inferred as tRNA-Trp [11], however, tRNA-Ser2 can be inferred instead with a slightly higher score (Additional file 5, 25). In this case, the ancestral gene order (*CytB*-Ser2) will be favored. The non-anticodon parts of this tRNA cannot be inferred unambiguously (Additional file 5, 23-25; Additional file 6, 4-5), although, because it is more consistent with that of other mites, the D-loop tRNA-Ser2 (D-loop) for *Steganacarus *is our preferred structure (Additional file 5, 25; Additional file 6, 5).

### tRNA-Cys

Dermauw et al. [12] inferred this tRNA as tRNA-Ala for *D. pteronyssinus*. Unfortunately, the 3' part of the acceptor stem of this putative structure is located inside a big, 30-nt deletion as compared with *D. farinae*. Thus, the same structure cannot be inferred for *D. farinae*. If the previous *D. pteronyssinus *reconstruction of tRNA-Ala [12] is entered into the program tRNAscan-SE, it suggests either tRNA-Ala (score 8.67) or Cys (TV-loop) (2.3). ARWEN suggests only tRNA-Cys (D-loop) (Additional file 5, 26). No similar analyses can be performed for *D. farinae *because of the deletion.

If flanking regions of the putative tRNA-Ala [12] are considered, tRNAScan-SE selects tRNA-Ala and, with a much higher score, tRNA-Cys (Additional file 5, 27-28). The position of tRNA-Ala is different between the two species (in *D. pteronyssinus*, it starts 1 nt downstream of the previous tRNA-Ala [12] and in *D. farinae *it overlaps with the acceptor stem of tRNA-Ser2 as inferred here). In contrast, the position of tRNA-Cys is the same in the two species, and this structure received the highest score, especially for *D. farinae *(18.8 vs. 4.98 for tRNA-Ala). ARWEN finds only this tRNA in both species (other tRNAs were also found but none of them is shared between the two species, Additional file 5, 27-28). tRNA-Cys is clearly more thermodynamically stable over tRNA-Ala (MFE negative vs positive in tRNA-Ala). For both species, BLAST search produced numerous significant alignments with the anticodon stem of tRNA-Cys of mites and other arachnids, crustaceans, and insects (Additional file 5, 27-28); for *D. farinae *only, BLAST also returned alignments with tRNA-Ala, but they were consistently worse than those with tRNA-Cys. In addition, alignment with four trombidiform species, for which the identity of tRNA-Cys is well established, clearly demonstrates a conserved pattern (three more matches in both the anticodon and D-stems, as compared to tRNA-Ala, see Additional file 7). We consider our ARWEN reconstruction of tRNA-Cys (Additional file 5, 27-28) as the most preferred based on its higher scores, the same position in two species, thermodynamics, similarities with GenBank data (especially with other acariform mites), and two putative compensatory mutations in the acceptor stem (in *D. farinae*, one of them occurring at the second position favoring tRNA-Cys over tRNA-Ala) and one such mutation in the anticodon stem (Fig. 2).

### tRNA-Pro

Our reconstructions agree with those of Dermauw et al. [12]. Interestingly, there is a difference how tRNAscan-SE and ARWEN treat the acceptor stem. The former program inferred it without a mismatch, while the latter one shifts its 5' end by one position upstream, creating one mismatch in both species. We present the structure consistent with tRNAscan-SE analysis as the preferred reconstruction (Fig. 2). The anticodon arm of this structure was confirmed by the BLAST similarity search (Additional file 5, 29-31). No tRNA-Pro [11] could be confirmed for *Steganacarus magnus*. This tRNA, as originally inferred, has an unusual anticodon (AGG not TGG), is thermodynamically unstable (positive MFE), and did not return any similarities with tRNA-Pro deposited in GenBank (Additional file 5, 32). In addition, no sequence conservation of the 3' part and the anticodon region is observed when aligned with *Dermatophagoides*, suggesting that this putative tRNA rather represents a random structure. In contrast, its 5' part displays apparent similarities with *Dermatophagoides *alignment and probably represents part of tRNA-Thr (see below).

### tRNA-Tyr (non-canonical)

Previously this tRNA was inferred as tRNA-Val [12] (= complement tRNA-Tyr). Unfortunately, both tRNAscan-SE and ARWEN could not confirm this structure (Additional file 5, 33), and there is strong evidence against it: (i) an indel occurs in the 5' part of the putative acceptor stem; (ii) the putative D-arm and acceptor stem have mismatches (1 and 2, respectively); (iii) if applied to *D. farinae*, the anticodon stem will have one mismatch.

In our analyses (Additional file 5, 34-40), tRNAscan-SE did not produce any structures, while ARWEN suggests various tRNA-Tyr for both *D. farinae *and *D. pteronyssinus*. Although the anticodon stem was the same among those tRNAs, there is a disagreement in the position of the acceptor stem:

(i) cloverleaf tRNAs-Tyr were found in both species (Additional file 5, 34-35; Additional file 6, 7). However, for *D. farinae *it was thermodynamically unstable (Additional file 5, 35). In addition, the indel is situated in the 5' part of the acceptor stems (Additional file 6, 1), this stem and T- and D-arms are not the same in the two species, and the acceptor and D-stems have mismatches. Although, mismatches in the acceptor stem can be edited in various organisms [7,20,21,33-35], this is not known for D- or T stems.

(ii) cloverleaf tRNA-Tyr (restricted). A separate search restricted to the conserved and apparently complementary regions including CCCTT(5') and GGGA(R)(3') returned another set of cloverleaf tRNAs (Additional file 6, 8-9). These tRNAs have negative minimum free energies and do not have the indel in their acceptor stem (Additional file 5, 37-38).

(iii) D-loop tRNAs. These tRNA were always preferred by ARWEN over the cloverleaf tRNAs based on a higher threshold value (95%, versus 90% and below for the cloverleaf tRNAs) (Additional file 5, 34-36). There is some disagreement between D-loop tRNAs; for example, in *D. pteronyssinus*, two different structures were inferred, and one of them was the same as the above cloverleaf tRNA except for the absence of D-arm (Additional file 5, 34), and it also included the indel. The other D-loop tRNAs do not include the indel in the acceptor stem, and its basic structure is shared among the two species, although the T-arms were not homologous, and the acceptor stem length was different (7 and 6-bp for *D. pteronyssinus *and *D. farinae*, respectively) (Additional file 6, 10-11). These structures, unlike the above cloverleaf tRNA have a negative free energy (Additional file 5, 35-36), indicating that they may be stable. The D-loop tRNA-Tyr is extremely rare in chelicerates, and it is only known for the following trombidiform mites: *Leptotrombidium *[9], *Walchia hayashii*, *Ascoschoengastia *sp., *Tetranychus urticae*, and *Panonychus ulmi*, but not for *Unionicola foili *where it is a TV-loop [10].

(iv) D-loop tRNAs (restricted). A separate search restricted to the conserved and apparently complementary regions including TAGACTTT(5') and AGGGTTTA (3') returned another set of D-loop tRNAs, very similar in both species (Additional file 5, 39-40). The differences involved the acceptor stem and the T-arm, inferred slightly differently. Fig. 2 shows the consensus between the two structures, which has a 7-bp acceptor stem and 2-bp T-arm. The consensus is very similar to the D-loop tRNA inferred in (iii).

(v) TV-loop tRNAs. This tRNA was inferred only for *D. farinae *(Additional file 5, 38), with a 4-bp D-stem having no mismatches and an unusually large, 15-nt, TV-loop (Additional file 6, 12). It has the lowest free energy value as compared to the above tRNA, but unfortunately this structure can be found only in this species. If strictly applied, the D-stem would have 3 mismatches in *D. pteronyssinus*.

Minimum free energy analyses found only one stable structure for each species. These structures have slight differences in their 5' ends, but the basic structure is the same: a long stem separated by 2 bulges at the 3' part, a loop, and a hairpin structure, representing the putative anticodon stem (Additional file 6, 13-14). These analyses favor the D-loop (analysis (ii) above) and cloverleaf tRNAs (iii).

At this point it is impossible to confidently select between the alternative structures, although we consider the D-loop tRNA (Fig. 2) as the preferred tRNA-Tyr (mostly based on its similarity with that of *Leptotrombidium*). As mentioned previously, the anticodon stem is the same across all structures, and it does produce many significant alignments with that of arthropods, indicating that our preliminary reconstructions may represent a functional tRNA, substantially deviating from the canonical tRNAs. It is interesting that in some other acariform mites (*Tetranychus urticae*, *Panonychus ulmi*) the canonical tRNA-Tyr (D-loop) cannot be inferred as well.

### tRNA-Lys

We confirm this tRNA, except for changing it from TV-loop to presumably cloverleaf tRNA (with the T-arm very short or it may be absent) and changing the putative 3' part of the acceptor stem of the previously established structure [12]. There is a 3-nt deletion in this region in *D. farinae*, therefore this putative acceptor stem is not likely to exist. In addition, this stem is only 6-bp long and has a mismatch (Additional file 5, 41). Our analyses suggest that the 3' part of the acceptor stem is located downstream (thus avoiding the deletion), its length corresponds to that of the canonical tRNA (7 bp), and it is fully complementary (with one compensatory mutation) (Additional file 5, 42-43). Our structures differ by only a small, 8-nt T-arm suggested by ARWEN for the two species (cloverleaf structure) (Fig. 2), while tRNAscan-SE does not infer it (TV-replacement loop). Since the ARWEN T-arm is too small, it may not exist in reality, however, the cloverleaf tRNA-Lys was described for trombidiform mites [10] and they also have a short (9-11 nt) T-arm. EST data, available only for *D. pteronyssinus *(e. g., EX162300 MW DP0257), in general favor our reconstruction over that reported previously [12], but the 3' end of our structure cannot be found unambiguously. In *D. pteronyssinus*, the polyadenylated tail is situated just before the 5'-end on the downstream tRNA-Asn (as predicted), and the predicted discriminator nucleotide of tRNA-Lys is situated at the n-1 position from the poly(A) site. In *D. farinae *there is a 2 nt deletion, so the predicted discriminator nucleotide overlaps with the first nt of tRNA-Asn (Fig. 2). This seems unlikely, but not impossible. Alternatively, the 3' end of both tRNAs would be at the n-2 position from the poly(A) site, but this would require a 6-bp acceptor stem.

We were able to find significant similarities of the anticodon stem of our tRNA-Lys (Fig. 2) with that of tRNA-Lys of *Atelura formicaria *(EU084035). *D. farinae *and *D. pteronyssinus *also display similarities with the anticodon stem of tRNA-Trp and non-anticodon fragments of others tRNAs (Additional file 5, 41-43). Interestingly, if the whole tRNA sequence is submitted, it will also align against *Ixodes *tRNA-Trp and other arthropods, and specifically to its anticodon stem. However, despite this significant hit the anticodon sequences are different between the two tRNAs. Because our tRNA is reconstructed with a high score, and tRNA-Trp was inferred confidently in another location (see below), we consider the similarities with tRNA-Trp as a result of convergent mutations constrained by the same secondary structure. Interestingly, a low score tRNA-Lys can be inferred at the beginning of putative *l-rRNA *of *Steganacarus magnus *(Additional file 5, 42-43).

### tRNA-Asn

We confirm this structure. Both tRNAscan-SE and ARWEN recovered this tRNA in *D. pteronyssinus *and *D. farinae *with high confidence values (Additional file 5, 45-46). There is no variation in any stem, except for the last nucleotide of the acceptor stem, where a non-compensatory mutation occurred in *D. farinae *(complementary in *D. pteronyssinus*). Identical mismatches were also found at its n-1 position and at the last position of the anticodon stem (Fig. 2). EST data [GenBank: EX162300MW DP0257] indicate that the 5' end of this tRNA is situated at precisely the same position as inferred. BLAST search of the anticodon stem returned significant similarities with tRNA-Asn of an insect and a spider (Additional file 5, 45-47).

### tRNA-Val (non-canonical)

The gene order *s-rRNA*-Val-*l-rRNA *(on the minority strand), is presumed to represent the ground pattern of the arthropod mitochondrial gene arrangement [41,42]. This gene order is not known for any acariform mite, however, in *Dermatophagoides*, the *s-rRNA *is followed directly by the *l-rRNA *(on the majority strand) without tRNA-Val in between [12]. Many arthropods also share the same absence of tRNA-Val but on the minority strand: certain Ricinuleida [43], Myriapoda [44], Crustacea: Malacostraca (with a non-coding region between the genes) [45], Ostracoda [46]; Insecta: Diptera [GenBank: NC_006378], Hemiptera [47], Phthiraptera [48]. Above we indicated that tentative tRNA-Val of Dermauw et al. [12] was probably inferred incorrectly and should be tRNA-Tyr. Although loss of tRNA-Val is not completely impossible, comparative analysis including three other astigmatid species (*Sancassania *sp., *Sturnophagoides bakeri*, *Gymnoglyphus longior*, *G. osu*) suggests that the region between *s-rRNA *and *l-rRNA *is conserved, and D-loop tRNA-Val can be inferred from it. The main difficulty with this tRNA concerns the extremely short (2 bp) T-stem and acceptor stem with only four paired bases (Fig. 2). No tRNA search program could confirm this structure, although they found similar structures (Additional file 5, 48-53). The corresponding sequence entered to mfold consistently renders a 6-bp "anticodon" stem and a 5-bp "acceptor" stem separated by a variable loop (Additional file 6, 15). However, mismatches in the acceptor stem can be edited after transcription [7,20,21,33-35], and the same short (2-4 bp) T-stem is known for trombidiform mites D-armless tRNAs [10]. The anticodon stem of this putative tRNA returns significant alignments with that of tRNA-Val of the crustacean *Argulus americanus *[GenBank: AY456187] and *Artemia franciscana *[GenBank: NC_001620.1], but hits on the start of *l-rRNA *and tRNA-Tyr were also present.

To address the question if our putative tRNA can be part of *l-rRNA*, a comparative set of 12 taxa was assembled and aligned using the reference structures of *Drosophila melanogaster *and *Caenorhabditis elegans *[49]. These taxa included: *Limulus polyphemus*, *Ixodes hexagonus*, *Habronattus oregonensis*, *Steganacarus magnus*, 3 species of *Leptotrombidium*, 2 species of *Dermatophagoides*, *Gymnoglyphus longior*, *Sturnophagoides bakeri*, and *Sancassania *sp. Unfortunately, because the 5' part of chelicerate *l-rRNA *(e. g., downstream of the conserved helix H563) is extremely variable in size among chelicerates, these data did not provide a definite answer regarding the relationships between *l-rRNA *and the putative tRNA-Val in Astigmata: (i) in the closest known outgroup (*Steganacarus*), the beginning of *l-rRNA *(as originally inferred) also has a distinct tRNA-like structure (tRNA-Lys) and this structure may represent an actual tRNA missed by the authors; (ii) in the second closest outgroup, *Leptotrombidium*, *l-rRNA *is 28 nt shorter than that of Astigmata (without the tRNA-Val like structure), which suggests that the putative tRNA-Val-like structure may exist as a tRNA and is not part of *l-rRNA*; (iii) *l-rRNA *of *Habronattus *is compatible to that of Astigmata (no putative tRNA-Val), indicating that astigmatid tRNA-Val may not be part of *l-rRNA*; (iv) *l-rRNA*s of *Limulus *and *Ixodes *are about 57 nt longer than the astigmatid *l-rRNA *(without the tRNA-Val-like structure) suggesting that this structure may be part of *l-rRNA*. In addition, EST data for *Dermatophagoides *from GenBank provide some evidence that tRNA-Val is not part *l-rRNA*: (i) 4 out of 21 ESTs cover the start of *l-rRNA *and none of them covers the presumed tRNA-Val region; (ii) one of these ESTs, covering most of the *D. farinae l-rRNA*, starts 3 nt downstream after the predicted end of the putative tRNA-Val. However, oftentimes ESTs do not cover starts of genes, so this evidence is very weak.

Because of the above preliminary arguments, we adopt the hypothesis that the conserved sequence at the beginning of astigmatid *l-rRNA *represents tRNA-Val, therefore the beginning of *l-rRNA *is truncated as described for *Habronattus *[22] and as evidenced from comparison with *Leptotrombidium*. This hypothesis is also supported by the seemingly homologous region downstream of *l-rRNA *helix H563, which usually has a 4-nt hairpin loop and the C- and G-rich 5' and 3' parts of the stem, respectively. In *Limulus *and *Ixodes*, this region corresponds to helix H461. If correct, 34 (out of 41) nucleotides between helices H533 and H461, as compared to *Limulus*, are missing in sarcoptiform mites and 24 such nucleotides are missing from the same region in *Habronattus*.

### tRNA-Trp

Both programs confirmed this tRNA, and its structure was identical. Some other tRNAs were inferred as well but none of them (except for tRNA-Phe) is shared between the two species. tRNA-Phe was identified between the end of *l-rRNA *and the start of tRNA-Trp, with a low cut-off value in *D. pteronyssinus *by ARWEN and in *D. farinae *by tRNAscan-SE with a low score (Additional file 5, 55-56). Because in both cases this putative structure was substantially worse than tRNA-Trp, we consider it as accidental. For *D. pteronyssinus *only, BLAST search returned numerous significant alignments with the anticodon stem of tRNA-Ser2(UCN) (which is complement of tRNA-Trp) with various arthropods (see above for BLAST searches of putative tRNA-Ser2, which was found similar to tRNA-Trp). Other evidence suggesting possible tRNA-Ser2 in this region is that this tRNA-Ser2 follows *ND1 *in the ancestral chelicerate genome, and the same pattern may be present here. However, inferring this tRNA as tRNA-Ser2 is not supported by the substitution pattern in *D. farinae*, where a mutation at the 4th position of the 3' part of the anticodon stem would be non-compensatory (Fig. 2), and, most importantly, the D-arm and the two adjacent 5' nucleotides of our structures retain the ancestral tRNA-Trp pattern (Fig. 2), which is also present in several trombidiform mites [10]. Furthermore, numerous EST data for both species [GenBank: CB284339DF1078, EX163125LY YIT DP1190] indicate the 5' end of this structure exactly as predicted by us for tRNA-Trp. BLAST search of the *D. farinae *anticodon arm sequence also confirms our inference (Additional file 5, 56).

It is interesting that inverted tRNA-Ser2 (D-loop) was found at the end of the large subunit ribosomal RNA in *Steganacarus magnus *[11], and its anticodon arm, acceptor stem, and putative D-arm have remarkable similarities with those of the complement of the putative tRNA-Trp of *Dermatophagoides *(Fig. 2). Most importantly, the evolutionarily conserved pattern described above is clearly recognizable (Additional file 6, 16, underlined), despite the putative D-arm (2 bp) having experienced severe reduction. ARWEN also suggested tRNA-Ser2 here (Additional file 5, 57), but it was less stable (MFE = -5.57 vs -8.45 for tRNA-Tyr), and the above similarities were no longer present. Based on these observations we believe that this is tRNA-Trp, not -Ser2 as it was suggested originally for *Steganacarus magnus *[11].

### Stem-loop Trp-ND1 structure/"tRNA-Tyr"

A highly unstable and divergent tRNA-Tyr was inferred for *D. pteronyssinus *[12]. In this structure, the anticodon stem has 2 mismatches, and the T-arm is only a 2-bp stem. The anticodon sequence, when aligned with *D. farinae*, suggests that the anticodon sequence in *D. farinae *is GUC, which corresponds to tRNA-Asp. In addition, the 3' part of the anticodon stem in *D. farinae *is further destabilized by two mismatches. Neither of the two tRNA-searching programs could confirm the structure of Dermauw et al. [12], however, tRNA-Ser1(AGN) was inferred by ARWEN for both species, but with a very low score (cut-off 70%) and a positive MFE (Additional file 5, 58-61). Furthermore, *D. pteronyssinus *EST data [GenBank: EX163586LY YIT DP1702] do not support the original tRNA structure because the polyadenylation site of the gene *ND1 *is situated inside the 3' part of the acceptor stem. BLAST search returned no significant alignment for this region, except for *D. farinae*, which returned hits of non-anticodon stems of tRNA-Arg (Additional file 5, 60). We refrain from inference of any tRNA, but a conserved stem-loop structure does exist here (Additional file 6, 17), indicating a possible biological function. Because this region is situated between two gene clusters transcribed on different strands: *COX1*-*l-rRNA *and *ND6-ND1*, this function is probably related to processing of the mRNA. See above for possible tRNA-Tyr inferred as tRNA-Val by Dermauw et al. [12].

### tRNA-Thr

*Dermatophagoides *retains the presumed ancestral chelicerate gene order "Phe-*ND5*-His-*ND4*-*ND4L*-Thr" in inverted form, and TV-loop tRNA-Thr was inferred in the corresponding sequence with a relatively high confidence (Additional file 5, 62-63). ARWEN suggested that this can be a cloverleaf tRNA-Cys in *D. farinae *(anticodon of this tRNA is complement of that of tRNA-Thr) (Additional file 5, 64), but this structure can be rejected because cloverleaf shaped tRNA-Cys is not typical of acariform mites in general and because the putative acceptor stem will be highly unstable if applied to *D. pteronyssinus*. If the sequence corresponding to the *D. pteronyssinus *tRNA-Thr is entered into ARWEN, nearly the same TV-loop tRNA is recovered (Fig. 2). The only difference between the two species is the D-stem, which has 4 and 3-bp in *D. pteronyssinus *and *D. farinae*, respectively. The D-stem does have the conserved pattern, GTT (5') and AA(3'), shared with trombidiform mites [10]. BLAST search returns many significant alignments of this tRNA with that of other arthropods, including mites (Additional file 5, 62-65). It is interesting that in *Steganacarus magnus *tRNA-Thr (cloverleaf or TV-loop) can also be inferred between *ND4L*-*ND6 *(ARWEN only) (Additional file 5, 66-67; Additional file 6, 19-20), and its putative anticodon stem has a significant similarity with that of tRNA-Thr of other arthropods (Additional file 5, 66-67) including, most importantly, acariform mites, *Dermatophagoides *(Additional file 8) and *Walchia hayashii *(NC_010595). This tRNA-Thr is situated between a non-coding region and *ND4L *in the acariform taxa, indicating that the region between *ND4L *and *ND6 *may be conserved at least in acariform mites; however, we refrain from giving a final structure of tRNA-Thr in *S. magnus *because of the following: (i) both cloverleaf and TV-loop (Additional file 6, 19-20) are thermodynamically unstable (MFE are 5.25 and 4.08, respectively), (ii) a stable stem-loop region can be inferred here (Additional file 6, 18). A highly unstable (MFE = 12.18) tRNA-Pro was proposed here following tRNAscan-SE [11]; it has 3 mismatches in the acceptor stem, 2 mismatches in the anticodon stem, which does not have any similarities with any known arthropod tRNA-Thr (Additional file 5, 66). We believe that the inference of tRNA-Pro in *Steganacarus magnus *represents a random structure rather than a tRNA.

### tRNA-His

This tRNA is also part of the presumed ancestral chelicerate gene order (see above for tRNA-Thr), and it can be inferred, with relatively high confidence, between the genes *ND5 *and *ND4*. Both programs give nearly the same structure consistent with that suggested for *D. pteronyssinus *[12]. ARWEN reconstruction gives a structure with an 8-bp acceptor stem, while for *D. pteronyssinus *the canonical 7-bp acceptor stem was inferred. tRNAscan-SE infers a 7-bp acceptor stem for both species. For *D. farinae*, we give the structure with a 7-bp acceptor stem (Fig. 2), which is consistent with ESTs data (5 sequences) unanimously suggesting a polyadenylation site immediately downstream of the 5' end of this structure. BLAST search returned numerous alignments with the anticodon stem of tRNA-His of insects, crustaceans, and arachnids (Additional file 5, 68-70), further confirming our inference.

### tRNA-Phe

This tRNA is also part of the presumed ancestral chelicerate gene order (see above for tRNA-Thr). This tRNA is unusual in *Dermatophagoides *in having a 6-bp anticodon stem (as opposed to 5-bp anticodon stem of the canonical tRNA) (ARWEN). A tRNAscan-SE reconstruction infers the anticodon stem starting at the same position but forces it to be 5-bp long, thus creating a non-canonical 9-nt anticodon loop (as opposed to 7 nt). At this point it is impossible to choose between the two possibilities (non-canonical stem or loop), Fig. 2 shows tRNA-Phe with 1^st ^5' nt of the non-canonical unpaired, making this stem canonical but suggesting 2 nt between the D- and anticodon arms (this may rarely occur elsewhere, for example in *Walchia *[10]). We compared this reconstruction with tertiary interactions proposed for tRNA-Phe of the nematode *Ascaris suum *[3]. The following base pairs were found: U^8^-A^14^, m^1^A^9^-A^23 ^(*D. pteronyssinus *only; m^1^A^9^-G^23 ^in *D. farinae*), G^10^-G^L2 ^become G^10^-A^L2 ^(both species), A^22^-A^L3 ^become A^22^-G^L3 ^(both species), U^15^-A^L4 ^become A^15^-U^L4 ^in *D. farinae *and U^15^-G^L4 ^in *D. pteronyssinus *(or, alternatively, this base pair does not exist), and A^26^-G^L1 ^become A^26^-A^L1 ^in *D. farinae *and G^26^-A^L1 ^in *D. pteronyssinus*. Interestingly, the D-arm of this tRNA retain the pattern GCTT (5') and AAGT (3') that seems to be conserved in trombidiform mites [10]. BLAST search retrieves significant alignments for the anticodon sequence with that of tRNA-Phe of chelicerates and sea spiders (Additional file 5, 71-73); sometimes searches also returned non-anticodon sequences of tRNA-Trp.

### tRNA-Ser1(AGN)

For both species, ARWEN inferred this tRNA as a D-loop (without a D-arm but with a T-arm) (Additional file 5, 78-80), which is the ground plan for metazoans [50,51]. For *D. pteronyssinus*, this tRNA was previously inferred with a 6-bp acceptor stem, 3-bp D-arm separated by an A, and lacking the variable loop [12]. ARWEN suggests a slightly different structure with 7-bp acceptor stem, a 3-bp D-arm (different from that of Dermauw et al. [12]) not separated from the acceptor stem (as in the canonical tRNA), and a 4-nt variable loop (Additional file 6, 21). This structure corresponds to that proposed for nematodes [6,38,39,52,53] and is adopted here. Unfortunately ARWEN could not recover the same structure for *D. farinae*, but instead it gave a 5-bp T-stem. We constrained this structure to that of *D. pteronyssinus *(Fig. 2). Significant positive alignment with the anticodon stem of tRNA-Ser1 was found for *Armillifer armillatus *(Pentastomida) [GenBank: NC_005934] only. It was identified as tRNA-Ser2 (anticodon complement of tRNA-Ser1) when aligned against the copepod *Paracyclopina nana *[GenBank: NC_012455]. In *D. farinae*, tRNA-Ser2 (TCN) was also inferred by tRNAscan-SE (TV-loop) and ARWEN (cloverleaf). Based on comparison with *D. pteronyssinus *and high MFEs (positive for the TV-loop and -1.65 for the cloverleaf), these reconstructions seem unlikely. MFEs for tRNA-Ser1 are -3.84 (*D. pteronyssinus*) and -6.93 (*D. farinae*).

### tRNA-Gln

Our reconstructions confirm this tRNA (Additional file 5, 81-83). The only difference was the ARWEN reconstruction of *D. farinae*, which has a 7-bp acceptor stem (as in the typical tRNA) and no overlaps (Fig. 2), while in *D. pteronyssinus *it has a 6-bp acceptor stem and 5' and 3' ends overlapping with tRNA-Ile and tRNA-Ser1, respectively by 1 nt. The D-arm has an evolutionarily conserved pattern, T.T (5') and A. (3'), as compared to trombidiform mites [10]. BLAST returned one positive alignment with the anticodon arm of the *D. pteronyssinus *sequence (which is 2-nt different from *D. farinae*). Other hits returned non-anticodon-arm fragments of tRNA-Trp or -His.

### tRNA-Ile

Dermauw et al. [12] inferred the tRNA-Ile and this was only confirmed by tRNAscan-SE for *D. farinae*. ARWEN suggests tRNA-Asp (D-loop) (which is complement to tRNA-Ile) in this position. Because the sequence of the putative anticodon arm returned multiple significant alignments with that of the tRNA-Ile of other arthropods (Additional file 5, 84-88), we performed ARWEN searches restricted to the opposite strand only. Results of the minimum free energy analyses unambiguously favor tRNA-Ile (MFEs range from -7.27 to -4.15) over tRNA-Asp (positive MFEs) (Additional file 5, cf. 86, 88 vs. 84-85, 87). A similar TV-loop structure was recovered for *D. pteronyssinus*, while for *D. farinae *the program suggested cloverleaf tRNA-Ile with a 2-bp T-stem. Since we expect tRNA-Ile to be a TV-loop, we constrained the *D. farinae *sequence to the tRNA-Ile of *D. pteronyssinus *(Fig. 2). It is very difficult to determine why both programs could not unambiguously infer this tRNA-Ile. In trombidiform mites, this tRNA was inferred as a D-loop [10].

### tRNA-Glu

We have additional sequence data for *Sturnophagoides bakeri*. In all three pyroglyphid species this tRNA was inferred as TV-loop tRNA-Glu, with a 5-bp anticodon stem, 4-bp D-stem and 6-7 bp acceptor stem (except for a 8-bp acceptor stem inferred with a mismatch for *D. pteronyssinus *by ARWEN) (Additional file 5, 89-92). The only difference from the previous reconstruction [12] is that the acceptor and D- stems (4, not 3 bp) were separated not by one (A) but 2 nucleotides (TA in all species), which is consistent with the canonical tRNA model. tRNAscan-SE suggests a 7-bp acceptor stem regardless of the mismatch at the beginning of the stem, while ARWEN predicts it as having 6 bp for most species (thus avoiding the mismatch). EST data strongly suggest that the acceptor step in fact has 7 bp [GenBank: EX162584MW DP0586, EX162102MW DP0015], thus indicating that the mismatch either is tolerated, or most probably, edited in the messenger RNA. Fig. 2 shows this reconstruction for *D. farinae *with a 7-nt acceptor stem. The acceptor stem has a number of compensatory mutations occurring at every position except for the 3rd position (Fig. 2) (or they can be explained by the 5' acceptor stem shift at the n+1 position with the concomitant shift of its 3' part to the n-1 position in *D. pteronyssinus*). *Dermatophagoides *shares the following conserved pattern with trombidiform mites [10]: CTT (5'), AAG (3') (acceptor stem); GT (5'), AAA, A. (3') (D-arm). GenBank data confirm this tRNA, and significant hits were especially numerous for the *D. farinae *anticodon arm sequence. However, the anticodon arm sequence of *S. bakeri *was aligned either with that of tRNA-Glu or tRNA-Phe(TTC)) (complement to tRNA-Glu(GAA)) (Additional file 5, 92).

### tRNA-Leu1(CUN)

We confirm this tRNA, except for the length of the D-arm that was inferred as having 3 bp [12]. In many of our analyses, all programs recovered essentially the same structure (4-bp D-arm and 7-bp acceptor stem as in the canonical tRNA); however, the following differences were found: D-arm has 3-bp (*D. pteronyssinus*, tRNAscan-SE only) and 8-bp acceptor stem (*D. pteronyssinus*, ARWEN). Here we give a structure with a 4-bp D-arm as suggested by the majority of analyses and a putative compensatory mutation (Fig. 2) for *D. farinae*. BLAST search also confirmed this tRNA (Additional file 5, 93-95). In contrast to the TV-loop tRNA-Leu1 of *Dermatophagoides*, this structure was inferred as either cloverleaf or D-loop tRNA in trombidiform mites [10].

### tRNA-Ala (non-canonical)

It was predicted as cloverleaf Cys [12], however none of the programs could confirm this or even converge on a single tRNA shared by both species (Additional file 5, 96-98). The original cloverleaf structure [12] disagrees in many respects with the canonical tRNA: the terminal base pair of the anticodon stem is unstable, the D-arm is separated by 2 nucleotides from the anticodon stem, and the acceptor and T stems have a mismatch. Unfortunately, in *D. farinae*, there is a large 14-nt deletion at the 3' part of the putative acceptor stem of *D. pteronyssinus *[12], thus the existence of this stem is highly unlikely. If flanking regions of the putative tRNA-Cys [12] are considered, the situation does not become clearer (Additional file 5, 97-98).

Unlike the above predictions, thermodynamic models of this region are very similar across the two species: a large hairpin structure with two stems separated by a connecting loop. One larger stem is 9-bp long has a single mismatch in the middle and another stem directly corresponds to the anticodon helix of tRNA-Ala (except for the stem being 6 bp long) (Fig. 2). The sequences of both stem regions are identical in both species, except for a single base pair in the 9-bp stem where compensatory mutations occur. If the search is restricted to this stable region then tRNA-Ala is consistently recovered in both species (inferring tRNA-Cys will create a mismatch at the last base pair of the anticodon stem) (Additional file 5, 99-100). Unfortunately, there is disagreement between these structures: in *D. farinae*, the acceptor and T-arms are situated at the n-1 positions, as compared to *D. pteronyssinus *(Fig. 2). All of these structures have positive MFEs, indicating that they may be unstable. Another structure, representing the full consensus between the two (with no mismatches) is possible, but it will have only a 7-8 bp acceptor stem, very short 6 nt T-arm (2 bp stem, 2 bp hairpin loop) and a 1-nt variable loop (Fig. 2), which is extremely unusual (only tRNA-Gln of *Walchia hayashii *presumably has a 7-nt T-arm and tRNA-Leu1 of *Unionicola foili *has 1 nt variable loop [10]). At this point it is impossible to infer confidently a canonical tRNA for both species in this region. We speculatively select the D-loop tRNA-Ala of *D. pteronyssinus *(Fig. 2) as the preferred structure, mostly based on its similarity with tRNA-Ala of trombidiform mites [10] and the fact that tRNA-Cys was inferred in another region with some confidence (see above).

BLAST search of the putative "anticodon arm" returned one significant alignment with a region located between the genes *ND3 *and *ND5 *and designated as "Non-translated intergenic spacer" of *Steganacarus magnus*. A cloverleaf tRNA-Ala could be inferred from it, with the anticodon stem, D-stem, and acceptor stems having 1, 1, and 2 mismatches, respectively. We refrain to infer this tRNA in *Steganacarus*.

### tRNA-Leu2(UUR)

We confirm this tRNA. Both tRNAscan-SE and AREWN inferred this structure as tRNA-Leu2 with high confidence. In one case, however, ARWEN suggested an 8-bp acceptor stem for *D. pteronyssinus*. It is also interesting that while *D. farinae *has the 7 canonical nucleotides in the anticodon loop (Fig. 2), *D. pteronyssinus *has 8 nt in this region. BLAST returned positive significant alignments with tRNA-Leu2 of various arthropods (Additional file 5, 101-103), but all of them have a 7-bp anticodon loop. Similarly to tRNA-Leu1 (see above), this tRNA-Leu2 in trombidiform mites has a typical cloverleaf structure [10], not a TV-loop as in *Dermatophagoides*.

### Polyadenylation sites mark 5' ends of tRNAs, discriminator nucleotides are not encoded

Three EST contigs, *COX3*-tRNA-Lys, *ND1*-*ND6*, and, on the opposite strand, *ND2*-Leu1, indicate the presence of polycistronic RNA units in *Dermatophagoides*. Although our data are incomplete, the distribution pattern of these RNA units suggests that transcription initiates at or close to the non-coding regulatory region of mtDNA. Processing of the majority of polycistronic units is believed to be nucleated by folding of the mt-tRNA structures [27], presenting a substrate for ribonuclease P, precursor tRNA 3'-endonuclease, and ATP(CTP)-tRNA-specific nucleotidyltransferase [54,55]. Different mitochondrial tRNA precursors are cleaved precisely at the tRNA 5' and 3' ends [54,56]. The implication of tRNA precursors in the processing of mRNA was first proposed for *Homo sapiens *[27] and then found in diverse organisms [31,57,58]. This may be a general mechanism of mRNA processing [59], although other mechanisms may exist [60], especially when all or most tRNA genes are absent from mitochondrial genomes [61,62].

The implication of the tRNA punctuation model of RNA processing is that the 5' end of a tRNA gene can be identified by a polyadenylated site in the mRNA. We found a pattern consistent with this model in *Dermatophagoides farinae *and *D. pteronyssinus*. Although available EST data are far from complete for either species, we were able to confirm the 5' ends of seven tRNAs using analyses of polyadenylated sites (-Asp, -Gly, -Asn, -Trp, -His, -Phe, and -Glu) (Fig. 2). In contradiction with this model of RNA processing [27], the discriminator nucleotide of 6 tRNAs (-Asp, -Gly, -His, -Glu, -Thr, -Leu2(UUR)) was found to overlap with the first position of a downstream gene (Fig. 2). Thus, our data indicate that (i) all tRNAs flanked by protein coding genes at both ends (-Asp, -Gly, -His, -Glu) follow the idea that precursor tRNAs are cleaved at exactly 5' end, and (ii) the putative 3' discriminator nucleotide overlaps with the first position of a translation initiation codon in these tRNAs (and also -Thr, Leu2). tRNA-Asn, -Trp, -Phe also follow pattern (i), but their 3' ends are flanked by non-protein coding regions: *l-rRNA*, untranslated region, and the D-loop, respectively; and another tRNA (-Ile) is expected to follow the same rule because its 5' end immediately follows the predicted 3' end of *ND2*. However, tRNA-Leu1(CUN), with its 5' end flanked by another tRNA, does not obey pattern (ii), and the presumed start codon of *CytB *is situated at the n+2 position from its 3' end.

Overlaps of the discriminator nucleotide with protein-encoding DNA as observed in *Dermatophagoides *are known [63-65], but they are not as widespread. Such overlaps with downstream tRNA genes are much more frequent. For example, they were recorded in annelids [66], crustaceans [67,68], insects [63], mammals [69], and birds [70]. In vertebrates it was demonstrated that this nucleotide is not encoded but added posttranscriptionally by polyadenylation [70,71], and we expect the same in *Dermatophagoides*.

### Posttranscriptional tRNA editing

In the previous section we suggested that the tRNA discriminator nucleotide at the 3' end is not encoded but added postranscriptionally at least in tRNA-Asp, -Gly, -Lys, -Thr, -His, -Glu, and Leu2(UUR). This was based on the fact that the predicted discriminator nucleotide was overlapping with the adjacent downstream gene. Other tRNAs may also be edited to add the discriminator nucleotide, but this needs to be shown experimentally.

With the exception of the suspected posttranscriptional addition of the discriminator nucleotide, there is an indication for posttranscriptional editing of the acceptor stem, as found in diverse organisms, including arthropods [7,20,21,33-35]. For example, mismatches at the beginning of the acceptor stem occur in 2 tRNAs (-Arg, -Val), at the end in 6 tRNAs (-Asp, -Cys, -Asn, -Phe, -Ser1(AGN), -Glu), and in the middle in 1 tRNA (-Ala) (Fig. 2).

U:U mismatches were also detected at the end of the acceptor stems in tRNA-Asn and -Glu. It is unknown if they are edited or tolerated.

## Conclusions

Mitochondrial tRNAs of *Dermatophagoides *are minimal, atypical tRNAs lacking either the T- or D-arm and thus deviate from the typical cloverleaf tRNA. The size of *D. farinae *tRNAs is 49-62, 54.4 ± 2.86 (range, mean ± SD) nt is significantly smaller than in *Caenorhabditis elegans *(53-63, 56.3 ± 2.30 nt) (p = 0.019) or *Ascaris suum *(51-62, 57.0 ± 2.67 nt) (p = 0.003), model minimal tRNA organisms. The shortest tRNA-Arg (49 nt) of *D. farinae *is similar to the shortest known tRNAs ranging from 45 to 49 nt [9,10]. In these extremely small D-armless tRNAs, T-stems are reduced to 2-3 bp and are thermodynamically unstable. Thus, this level of simplicity approaches that of the original adaptor RNA envisioned by Francis Crick [72], raising the question if a tRNA lacking both D- and T-arm may exist. Certainly experimental data are needed to further investigate this interesting issue.

Inference of minimal tRNAs is difficult because tRNA search programs do not incorporate models of extremely short tRNAs of acariform mites, and the acceptor stem may have mismatches, which are later edited postranscriptionally. Based on various lines of evidence, including polyadenylation and BLAST searches restricted to anticodon arms, we amend the identity of four tRNAs in *Dermatophagoides *(tRNA-Cys, -Tyr, -Val, -Ala) and propose a different secondary structure for another six (tRNA-Arg, -Ser2, -Lys, -Ser1, -Glu, -Leu1) (Table 1, column "Prev"). Most notably, two previously inferred cloverleaf structures and another extremely short and unstable structure were not supported by our data. Thus, widespread occurrence of the remarkably short, non-cloverleaf tRNAs are characteristic of all acariform mites known to date.

Furthermore, we found evidence that in *Dermatophagoides*, tRNAs may serve as processing signals for polycistronic mt RNA transcripts, the tRNA discriminator nucleotide is not encoded and added postranscriptionally, and that mismatches in the acceptor stem are probably indicative of their posttranscriptional editing.

## Methods

### Mite strain and DNA extraction

Mites were obtained from a laboratory culture maintained at the University of Michigan, Museum of Zoology (BMOC 05-0812-001) started from specimens collected in a skeleton-cleaning culture of *Dermestes maculatus *(Coleoptera: Dermestidae) in Ann Arbor, MI in 2005. Genomic DNA was isolated from a single individual in 5 replicates using the QIAamp^® ^DNA Micro (Qiagen), with the manufacturer's protocol for tissues modified as follows: a) the mite was crushed or pierced with a sterile pin in a drop of buffer ATL before transferring to 180 μL of buffer ATL (step 1 in the manufacturer's protocol); b) Proteinase K was added but not mixed (step 3); c) the incubation time (proteinase K lysis) was extended to 24 hr (step 4); d) no carrier RNA was added (step 5); e) DNA bound to the silica-gel membrane was eluted in 30 μL of buffer AE. Slide-mounted mites were identified using morphology [73], and the identification was confirmed by GenBank EST sequences for multiple mitochondrial genes.

### Amplification and sequencing

A series of nested PCRs using general mite or acariform mite-specific degenerate oligonucleotide primers (Additional file PCR Primers) was performed to amplify and sequence 4 gene fragments (*COX1*-*COX2*, *s-rRNA*-*l-rRNA*, *ND4*-*ND5*, *CytB*). The sequences obtained were used to design 8 species-specific oligonucleotide primers (forward and reverse for each gene fragment) for a long PCR (Additional file 9). A total of 28 long PCRs using all possible combinations of specific primers were run using High Fidelity Platinum^® ^Taq. This resulted in four positive amplicons spanning almost the entire mitochondrial genome, except for *ND2 *and the D-loop (control region). These amplicons were sequenced via primer walking. We performed several hundred reactions with additional primers targeting the missing region, but all of them were unsuccessful. Sequence data from the recently published mitochondrial genome of *Dermatophagoides pteronyssinus *[12] and the use of Expand Long Range polymerase from Roche [12] helped us to amplify the missing region. The D-loop of *D. farinae *contained two long AT repeats (one in *D. pteronyssinus*), effectively inhibiting all our previous long PCRs. Because this region had a variable length and could not be sequenced directly, a 0.95 kb PCR amplicon was cloned (TOPO^® ^TA Cloning^® ^Kit), and six clones, selected to include all the observed extremities in size, were sequenced [GenBank: GQ465337-GQ465342]. Sequence of the whole mitochondrial genome of *D. farinae *was annotated to reflect proposed changes in gene regions and include EST data [GenBank: GQ469891]. Primers were designed in Primer3 [74].

The 20-μL PCR mix contained 2.0 μL 10 × PCR buffer, 1.4 μL of 50 mM MgSO_4_, 1.4 μL of dNTPs (10 mM each), 0.8 μL each of 10 μM primer, 0.08-0.12 μL of polymerase, and typically 0.3 μL of DNA template (not quantified). For regular PCRs, Platinum^® ^Taq was used, whereas for long PCRs a mix of this and High Fidelity Platinum^® ^Taq at the proportion of 1:1.8 was used. For the Expand Long Range and Expand Long Template polymerases, the manufacturer's protocol was followed, with the addition of DMSO (final concentration 3 mM). Long PCR cycling conditions were as follows: 94°C for 1:50 min; 94°C for 0:30; 50°C for 0:35, 10 cycles at 58-68°C for 2:00-12:00 (depending on experiment); 25 cycles with the extension time increased by 2-5 s/cycle, other parameters are the same; 58-68°C for 7:00-12:00. Regular PCRs were run with the extension temperature of 72°C, usually without the extension time increment. All reagents, unless otherwise specified, were from Invitrogen Corporation (USA). PCR products were visualized on 1.5% agarose gels, purified using QIAquick Gel Extraction Kit (Qiagen), and sequenced in both directions by the University of Michigan DNA Sequencing Core on an Applied Biosystems 3730 DNA Analyzer. Sequences were assembled in Sequencher 4.9 (Gene Codes Corporation, Ann Arbor, Michigan).

### Detection of tRNAs

The web version of the program tRNAscan-SE [18] and a standalone version of the program ARWEN [19] were used to detect tRNAs and infer their secondary structure. For tRNAscan-SE, the following parameters were changed: organism = "Nematode Mito", origin = yes, ace = yes; fops = yes, breakdown = yes, gcode ="Invertebrate Mito", covescore = 0.1, euparams = relaxed. These setting were saved in a custom web form to unsure uniform searches http://insects.ummz.lsa.umich.edu/ACARI/tools/tRNAscan-SE/. ARWEN was run with the following parameters: -l -seq -gcinvert -br and variable threshold parameter -ps (70, 80, 90, 95, and 100). To choose between alternative structures from tRNAscan-SE and ARWEN, minimum free energy (MFE) was calculated for these structures (constrained analysis), as well as for the primary sequence (unconstrained analysis) in mfold [75]. Secondary structure information was converted to the mfold and XRNA format and then submitted to mfold using a custom javascript function embedded in the webpage http://insects.ummz.lsa.umich.edu/ACARI/tools/ARWEN_to_mfold.

Because *Dermatophagoides *has atypical tRNAs lacking either the D- or T-arms, BLAST similarity search of the whole tRNA sequence was usually not informative. Therefore, we restricted our searches only to the anticodon arm, which is a less variable sequence as compared to the canonical tRNA. Additional filters were enabled to restrict searches to non-coding regions of arthropod mitochondrial genomes http://insects.ummz.lsa.umich.edu/ACARI/tools/BLAST_custom.htm. Available GenBank EST sequence data were used to confirm the 5' end of several tRNA [27].

We obtained sequences of tRNA-Val (located between l-and *s-rRNA*) for an additional 4 species (*Gymnoglyphus longior *[GenBank: GQ465344], *G. osu *[GenBank: GQ465345], *Sturnophagoides bakeri *[GenBank: GQ465343], *Sancassania sp*. [GenBank: GQ465346]), and for tRNA-Glu for one species (*Sturnophagoides bakeri *[GenBank: GQ465347]). Alignment of tRNAs is available in Additional file 10. tRNAs were visualized in the program XRNA.

## Abbreviations

A: adenine; *ATP6 *and *8*: ATPase subunit 6 and 8; bp: base pairs; C: cytosine; *COX1*-*3*: cytochrome oxidase subunits I-III; *CytB*: cytochrome b; D-arm: dihydrouridine-arm of a tRNA secondary structure; D-loop tRNA: (D-arm)-replacement loop tRNA; D-loop: displacement loop (mitochondrial control region); EST: expressed sequence tag; G: guanine; L2 = UUR; *l-rRNA*: large (16S) rRNA subunit gene; MFE: minimum free energy; mRNA: messenger RNA; mt: mitochondrial; *ND1-6 *and *ND4L*: NADH dehydrogenase subunits 1-6 and 4L; nt: nucleotides; PCR: polymerase chain reaction; rRNA: ribosomal RNA; S1 = AGN; S2 = UCN), transfer RNA; *s-rRNA*: small (12S) rRNA subunit gene; T: thymine; T-arm: TΨC-arm of a tRNA secondary structure; tRNA-X: (where X is replaced by a one letter amino acid code for the corresponding amino acid, with L1 = CUN; TV-loop: (T-arm and variable loop)-replacement loop tRNA.

## Authors' contributions

PBK conceived of the study, carried out the molecular genetic studies, participated in the sequence alignment, performed the statistical analysis, and drafted the manuscript. BMOC participated in the design of the study and drafted the manuscript. All authors read and approved the final manuscript.

## Supplementary Material

Additional file 1 **Nucleotide composition and GC and AT skews of protein-coding genes on the majority strand of *Dermatophagoides farinae***. Analysis of nucleotide composition and GC and AT skews for 13 protein-coding genes. For each gene, values are given for all sites and for 4-fold degenerate sites only. Four scatterplots summarize the data. Click here for file

Additional file 2 **Codon usage and relative synonymous codon usage (RSCU) values for mitochondrial proteins of *Dermatophagoides farinae***. Values of codon usage (per thousand) and relative synonymous codon usage are recorded for each position and strand (with a statistical test for significant strand bias). Data are represented in tables and histograms on multiple worksheets. Click here for file

Additional file 3 **Nucleotide composition, strand bias and annotation of protein coding genes and the control region (D-loop) in the mitochondrial genomes of *Dermatophagoides *spp**. Describes nucleotide composition and strand bias in *D. farinae*. Also includes a comparative analysis of *Dermatophagoides farinae *and *D. pteronyssinus *mitochondrial genes (except for tRNAs) with particular emphasis on features which were previously inferred incorrectly. Click here for file

Additional file 4 **Secondary structures forming between genes *ND6 *and *ND1 *in *Dermatophagoides *spp**. Stem loop structures present between ND6 and ND1 genes in *Dermatophagoides farinae *and *D. pteronyssinus*. Click here for file

Additional file 5 **Transfer RNAs in *Dermatophagoides *spp. and some other acariform taxa**. Analyses of transfer RNAs of *D. farinae *and *D. pteronyssinus *and some other related taxa using tRNAscan-SE and ARWEN. For each putative tRNA found, BLAST similarity search is performed (anticodon arm only) and minimum free energy values (mfold) are recorded. Click here for file

Additional file 6 **Transfer RNAs and minimum free energy structures in *Dermatophagoides *spp. and *Steganacarus magnus***. Transfer RNAs of *D. farinae *(DF), *D. pteronyssinus *(DP), and *S. magnus *(SM) found by tRNAscan-SE and ARWEN and alternative free energy structures inferred by mfold. Putative compensatory mutations are indicated by an asterisk. Click here for file

Additional file 7 **Alignment of tRNA-Cys of *D. farinae *and five other acariform mites**. Secondary structure information is indicated by square brackets. Conserved sequence motif is indicated by asterisks. tRNA-Ala of *Leptotrombidium pallidum*, which is very similar but clearly distinct from tRNA-Cys, is also given for comparison. Best viewed in MacClade or Mesquite http://mesquiteproject.org/. Click here for file

Additional file 8 **Alignment of the region between genes *ND6 *and *ND4L *for *Dermatophagoides *spp. and *Steganacarus magnus***. Secondary structure information of tRNA-Thr is indicated by parenthesis, anticodons are color-coded (blue *Dermatophagoides *spp.; red *S. magnus*). The alignment suggests that the original tRNA-Pro of *S. magnus *was probably inferred incorrectly. Click here for file

Additional file 9 **General oligonucleotide primers used to amplify four gene fragments of *Dermatophagoides farinae *mitochondrial genome**. Primer sequences are written from the 5' end. Click here for file

Additional file 10 **Annotated alignment of *D. farinae *mitochondrial genome**. Annotated alignment of *D. farinae *mitochondrial genome with other taxa, including secondary structure annotations for *s-rRNA*, *l-rRNA*, and tRNAs, and polyadenylation sites mapping. Best viewed in MacClade or Mesquite http://mesquiteproject.org/. Click here for file
