# Supplementary material for: Improved tRNA prediction in the American house dust mite reveals widespread occurrence of extremely short minimal tRNAs in acariform mites
Source: BMC Genomics. 2009 Dec 11;10:598. doi: 10.1186/1471-2164-10-598 (PMC2797822; doi:10.1186/1471-2164-10-598)
Supplement: Additional file 3 — Nucleotide composition, strand bias and annotation of protein coding genes and the control region (D-loop) in the mitochondrial genomes of Dermatophagoides spp. Describes nucleotide composition and strand bias in D. farinae. Also includes a comparative analysis of Dermatophagoides farinae and D. pteronyssinus mitochondrial genes (except for tRNAs) with particular emphasis on features which were previously inferred incorrectly. [file 1471-2164-10-598-S3.DOC]

## Nucleotide composition and strand bias

*Dermatophagoides farinae* mitochondrial DNA displays a positive GC-skew (0.2314) and a negative AT-skew (-0.2531), i.e. the transcript of the major strand is relatively rich in nucleotides G and T, and correspondently poor in C and A (nucleotide frequencies as follows: T 0.447, C 0.110, A 0.267, G 0.176). This pattern, referred to as the reverse strand bias [1], is also found in nematodes, flatworms, lancelets, several crustaceans, insects, and arachnids, including *Dermatophagoides pteronyssinus* [1]. In contrast, negative GC-skew and positive AT-skew is common for vertebrates and echinoderms, except from *Florometra* [1-5]. In vertebrates, strand bias directly reflects the difference in molecular weight of the two strands of the mitochondrial DNA, which are termed heavy and light. The G-rich heavy strand encodes the majority of genes. Its transcript is, thus, G-poor and C-rich (negative GC-skew and positive AT-skew). During replication, the light strand is the leading strand, and it remains double stranded, while the heavy strand is a lagging strand, which remains single-stranded for a long time, and thus, more vulnerable to damage by the nonenzymatic methylations, oxidation, and hydrolic deamination [6]. Mutations from adenine to hypoxanthine leading to accumulation of Gs on the lagging strand may be especially frequent [7]. Since synthesis of the leading light strand, which pairs to the parental single-stranded heavy strand, starts at a specific place (replication origin of the light strand, O_L_), the more a H-stranded gene is close to O_L_ the less pronounced the bias created by asymmetric replication is. For example, the G-content of the lagging H-strand increases from O_L_ to O_H_ (origin of the heavy strand), while in the transcript from this strand, it will inversely decrease due to complementarity as observed for mammals [6, 7]. The strand bias also affects the aminoacid composition of mitochondrial proteins, causing significant artifacts in phylogenetic reconstructions utilizing a mixture of taxa with the normal and reverse strand biases [7]. Recent studies cast doubt on the orthodox strand displacement mode replication described above and suggest that mtDNA replication proceeds mainly, if not exclusively, by a strand-coupled mechanism [8-10]. Review of both models of DNA replication with their supporting evidence has recently been published in a series of arguments [11-14]. It is interesting that in insects the replication origins of both strands (the leading strand is termed the minor and the lagging strand is termed the major coding strand) are located in the D-loop (control region). Thus, nearly 97% of the lagging strand of insects may remain single-stranded during replication, in contrast to only 2/3 of the lagging strand being single-stranded in mammals [15]. Despite this, some insects, such as *Drosophila*, do not show a distinct strand bias [2], suggesting a mechanism counterbalancing the consequences of the possible asymmetric nature of mitochondrial DNA replication. In *Drosophila melanogaster*, G to A mutations on the sense strand were experimentally shown to be the most frequent mutations, possible accounting for to the extreme high AT content on both strands observed in this species [16].

As indicated above, there is a strong strand bias in *D. farinae* and it even more pronounced as compared to *D. pteronyssinus*. The majority strand displays the reverse pattern (e. g., positive GC-skew and negative AT skew) to what observed in mammals. This pattern was found for all gene regions, except for *ATP8* and *ND1*. Both GC and AT skews of *ATP8* are negative but the GC skew is close to zero (Additional file 1). *ND1* is situated on the minority strand and its GC and AT skews a nearly the same and approaching zero, as on would expect from genes without asymmetrical mutational bias. It is interesting that patterns of GC- and AT-skews as well as nucleotide compositions is essentially the same when considered overall or only for 4-fold degenerate sites (Additional file 1), indicating that the asymmetric strand bias profoundly affects aminoacid composition in this mite species as reported previously for flatworms [3], and may be source of significant artifacts in phylogenetic interference. There is a clear preference for GT-rich codons on the majority strand, while AC-rich codons were more frequent on the minority strands, however, only at the third position the differences were statistically significant. We were unable to detect any coherent pattern indicating the origin of the leading or lagging strand synthesis as it was done for mammals [6]. However, this pattern may be compromised by frequent gene inversions as compared to ancestral chelicerate ground plan or differences in mitochondrial DNA replication, which is not characterized in chelicerates. Overall cumulative relative synonymous codon usage values (RSCU) [17] were not significantly different across the two strands (p=0.9974), however, there was a clear significant bias at the third position. The majority strand had more G and T at the third position (p<0.001), while the minority strand has more A and C (p<0.001). Codon usage along with RSCU values are given for each gene in Additional file 2.

## COX1 start codon

The COX1 start codon of *D. pteronyssinus* was inferred as ATA [5]. There is a non-start codon (GGA) at this position in *D. farinae*, while both species have the same start codon at 3-nt upstream, ATT. We believe that the start codon of *COX1* is situated at this position.

## ND3 start codon

The translation initiation codon as inferred previously [5] makes the beginning of the resultant protein very short compared to GenBank data [GenBank:YP_073289, YP_002317251, YP_054511, YP_448913, NP_071844, CAC69942, YP_002640593]). There is a start codon (TTG) that is shared by the two species 12 nt upstream from the previously inferred position [5], and it is the same as the discriminator nucleotide (the 3’ dangling end) of tRNA-Gly. The pattern of a tRNA discriminator nucleotide is common in the *D. farinae* mitochondrial genome. In addition, our inference of the start codon for ND3 makes this protein fully compatible with that of *Ixodes uriae* and more compatible with those of other arthropods (2-8 aa longer).

## ND3 stop codon

No shared full stop codon can be found in the two species. In *D. pteronyssinus*, it was inferred as TAA, and as TAG, situated 3 nt downstream in *D. farinae* [5]. Apparently, there is also the same “codon” TAG in *D. pteronyssinus*, preceded by a 1nt insertion causing a frame shift. For both species this inference is contradicted by GenBank protein sequences, which end downstream. At the end of *ND3* GenBank protein sequences, there is a conserved amino acid W (Trp) usually followed by 2 less conserved amino acids downstream. An inference of a stop codon at the conservative end of ND3 would be problematic because the first nt here is an A, thus a functional stop codon cannot be formed by polyadenylation. The first nucleotide at the downstream n-3 position is a T (so the stop codon can be completed by addition of two A residues to the mRNA) and it is the beginning of our putative tRNA-Arg (however, the inferred beginning of this tRNA overlaps by 1 nt with the end of ND3; the tRNA punctuation model of RNA processing [18] dictates that the 5’ end of a tRNA immediately follows the 3’ end of a RNA coding sequence). This is our preferred preliminary hypothesis regarding the end of *ND3*; the same end of *ND3* is known for *Pachypsylla venusta* [GenBank:YP_073289].

## ND6 start codon

ATG is suggested to be the start codon for *D. pteronyssinus*. Unfortunately, in *D. farinae* there is a non-start codon (TCA) at this position. Generally, it is very difficult to find a start codon based on comparison with other mites because the end of *ND6* is very variable in terms of amino acid pattern and length, with *Steganacarus magnus* being the longest and *Tetranychus urticae* the shortest. These unalignable stretches usually contain many potential start codons. For example, there are five such codons in *D. farinae*. Given that the true start codon is impossible to infer objectively, we consider TTG situated 15 nt upstream of the *D. pteronyssinus* start codon as inferred previously [5]. If true, *ND6* is started at the discriminator nucleotide of tRNA-Thr (3’ end of the tRNA), and no non-coding regions exist between tRNA-Thr and *ND6*.

## ND6 stop codon

TAA was suggested as the stop codon for *ND6* of *D. pteronyssinus* [5], making it one of the longest mite *ND6* sequences. The sequences of the two *Dermatophagoides* species are unalignable as amino acids at 9 nt stretch upstream of the putative stop codon of *D. pteronyssinus*. Given this, we believe that the *ND6* stop codon is the first T in this region, making the length of sequences of both species compatible with those established for other mites; for example, it exactly matches the end of *ND6* in *Carios capensis* and *Ixodes holocyclus*. In both *D. pteronyssinus* and *D. farinae*, it is preceded with the codon TAT encoding amino acid tyrosine. EST data fully support our inference, clearly showing that the partial stop codon (as inferred by us) is complemented by a polyadenylated tail in the mRNA (EX162204 MW DP0138).

Alternative polyadenylation is found in *D. pteronyssinus* [GenBank:EX163678]. The alternative poly(A) is located 20 nt downstream of our stop codon and includes a sequence for translational termination codon (TAA) as suggested previously [5]. As noted above, no similar termination codon was found in *D. farinae*. There are two possible explanations for this phenomenon: (i) the *ND6* transcript matures in several steps; EX163678 represent an intermediate step, whereas another EST [GenBank:EX162204] represent the final step in both species; (ii) alternative polyadenylation (EX163678) may extend the mRNA code for a different protein in *D. pteronyssinus* only. Here we adopt the more conservative first hypothesis with the warning that more data are necessary to elucidate this interesting issue.

The sequence between the end of *ND6* (as inferred here) and ND1 is 59 and 39-nt long in *D. farinae* and *D. pteronyssinus*, respectively. Its 5’ and 3’ ends form a distinct 5-nt stem in both species, and it has a poly-C region (Additional file 4). Probably it serves as mRNA processing signal.

## ND5 start codon

The *ND5* start codon of *D. pteronyssinus* was inferred as ATT with a 5-nt non-coding region upstream [5]. There is a non-start codon (GTT) at this position in *D. farinae*, while both species have a start codon at 6-nt upstream, ATA in *D. farinae*, and TTG in *D. pteronyssinus*. We believe that the start codon of *ND5* is situated at this position.

## D-loop

We were unable to detect any pattern consistent with those described for insects [19, 20], mammals [21, 22], or vertebrates [23]. In *Dermatophagoides*, the putative D-loop is flanked by tRNA-Phe at the 5’ end and by tRNA-Ser1 at the 3’ end. Additional file 10 presents an annotated alignment, describing features of the D-loop regions of *D. farinae* and *D. pteronyssinus*. In *D. farinae* only, at the 5’ end of this region, there is a distinct T-shaped structure, which is very similar to cruciform/tRNA-like structures known in mammals and birds [23, 24]. This structure is absent in *D. pteronyssinus*, indicating that it may represent a random sequence of no functional significance. There is an AT-repeat downstream of this structure in *D. farinae* and downstream of the 3’ end of tRNA-Phe in *D. pteronyssinus*. In the former species it can be 24-58 nt in length in a single individual indicating the presence of heteroplasmy, while in the latter it summarily measures 14-56 nt for about 1000 specimens [5]. Immediately downstream of this repeat, an AT-rich region follows, which also contains a short AT repeat (8 nt) in *D. farinae*. Downstream of this region, a stable hairpin structure (15 bp stem, 7 nt loop) is situated, corresponding to the terminal stem of structure #2 in *D. pteronyssinus* [5]. Based on the presence of two short palindromic sequences, TACAT and ATGTA, also found in fishes and mammals, this stem was attributed to the stem-loop structure of the L-domain [5], which might serve as the recognition site for the arrest of J-strand synthesis [22]. These sequences are absent in *D. farinae*, but the structure of the stem was, nevertheless, maintained by compensatory mutations. Out of seven stem-loop structures proposed for *D. pteronyssinus* [5], only this and structure #6 (situated downstream) are supported by our data. Structure #6 has a 20-bp stem with a conserved connecting loop (9 nt) at the 5’ part and a variable hairpin loop. Between structure #6 and the terminal stem of structure #2, there is another large (34-38 nt) AT-repeat in *D. farinae*. In *D. pteronyssinus*, it is represented by an AT-rich region. The region between the stem #6 and the 3’ of the putative D-loop has low sequence conservation between the two species.

In conclusion, the consensus of D-loop structure between the two species is as follows (ncsb=non-conserved sequence block): ncsb1 - variable length AT-repeat (24-58 nt in *D. farinae*) - hairpin stem (15 bp stem, 7 nt loop) - variable AT-rich ncsb (52-56 nt in *D. farinae*, 38 nt in *D. pteronyssinus*) - ncsb2 - 20-bp stem with a conserved connecting loop (9 nt) at the 5’ part and a variable hairpin loop (3 nt in *D. farinae* and 6 nt in *D. pteronyssinus*)- ncsb3.

# References

1. Hassanin A: **Phylogeny of Arthropoda inferred from mitochondrial sequences: Strategies for limiting the misleading effects of multiple changes in pattern and rates of substitution**. *Mol Phylogenet Evol* 2006, **38**(1):100-116.

2. Perna NT, Kocher TD: **Patterns of nucleotide composition at fourfold degenerate sites of animal mitochondrial genomes**. *J Mol Evol* 1995, **41**(3):353-358.

3. Min XJ, Hickey DA: **DNA asymmetric strand bias affects the amino acid composition of mitochondrial proteins**. *DNA Res* 2007, **14**(5):201-206.

4. Hassanin A, Leger N, Deutsch J: **Evidence for multiple reversals of asymmetric mutational constraints during the evolution of the mitochondrial genome of Metazoa, and consequences for phylogenetic inferences**. *Syst Biol* 2005, **54**(2):277-298.

5. Dermauw W, Van Leeuwen T, Vanholme B, Tirry L: **The complete mitochondrial genome of the house dust mite, *Dermatophagoides pteronyssinus* (Trouessart): a novel gene arrangement among arthropods**. *BMC Genomics* 2009, **10**(1):107.

6. Reyes A, Gissi C, Pesole G, Saccone C: **Asymmetrical directional mutation pressure in the mitochondrial genome of mammals**. *Mol Biol Evol* 1998, **15**(8):957-966.

7. Faith JJ, Pollock DD: **Likelihood analysis of asymmetrical mutation bias gradients in vertebrate mitochondrial genomes**. *Genetics* 2003, **165**(2):735-745.

8. Yang MY, Bowmaker M, Reyes A, Vergani L, Angeli P, Gringeri E, Jacobs HT, Holt IJ: **Biased incorporation of ribonucleotides on the mitochondrial L-strand accounts for apparent strand-asymmetric DNA replication**. *Cell* 2002, **111**(4):495-505.

9. Bowmaker M, Yang MY, Yasukawa T, Reyes A, Jacobs HT, Huberman JA, Holt IJ: **Mammalian mitochondrial DNA replicates bidirectionally from an initiation zone**. *J Biol Chem* 2003, **278**(51):50961-50969.

10. Yasukawa T, Reyes A, Cluett TJ, Yang MY, Bowmaker M, Jacobs HT, Holt IJ: **Replication of vertebrate mitochondrial DNA entails transient ribonucleotide incorporation throughout the lagging strand**. *EMBO J* 2006, **25**(22):5358-5371.

11. Brown TA, Cecconi C, Tkachuk AN, Bustamante C, Clayton DA: **Replication of mitochondrial DNA occurs by strand displacement with alternative light-strand origins, not via a strand-coupled mechanism**. *Genes Dev* 2005, **19**(20):2466-2476.

12. Bogenhagen DF, Clayton DA: **Concluding remarks: The mitochondrial DNA replication bubble has not burst**. *Trends Biochem Sci* 2003, **28**(8):404-405.

13. Bogenhagen DF, Clayton DA: **The mitochondrial DNA replication bubble has not burst**. *Trends Biochem Sci* 2003, **28**(7):357-360.

14. Clayton DA: **Mitochondrial DNA replication: What we know**. *IUBMB Life* 2003, **55**(4-5):213-217.

15. Saito S, Tamura K, Aotsuka T: **Replication origin of mitochondrial DNA in insects**. *Genetics* 2005, **171**(4):1695-1705.

16. Haag-Liautard C, Coffey N, Houle D, Lynch M, Charlesworth B, Keightley PD: **Direct estimation of the mitochondrial DNA mutation rate in *Drosophila melanogaster***. *Plos Biology* 2008, **6**(8):1706-1714.

17. Sharp PM, Tuohy TMF, Mosurski KR: **Codon usage in yeast - cluster analysis clearly differentiates highly and lowly expressed genes**. *Nucleic Acids Res* 1986, **14**(13):5125-5143.

18. Ojala D, Montoya J, Attardi G: **Transfer RNA punctuation model of RNA processing in human mitochondria**. *Nature* 1981, **290**(5806):470-474.

19. Zhang DX, Hewitt GM: **Insect mitochondrial control region: A review of its structure, evolution and usefulness in evolutionary studies**. *Biochem Syst Ecol* 1997, **25**(2):99-120.

20. Zhang DX, Szymura JM, Hewitt GM: **Evolution and structural conservation of the control region of insect mitochondrial-DNA**. *J Mol Evol* 1995, **40**(4):382-391.

21. Sbisa E, Tanzariello F, Reyes A, Pesole G, Saccone C: **Mammalian mitochondrial D-loop region structural analysis: identification of new conserved sequences and their functional and evolutionary implications**. *Gene* 1997, **205**(1-2):125-140.

22. Saccone C, Pesole G, Sbisa E: **The main regulatory region of mammalian mitochondrial DNA: structure-function model and evolutionary pattern**. *J Mol Evol* 1991, **33**(1):83-91.

23. Brown GG, Gadaleta G, Pepe G, Saccone C, Sbisa E: **Structural conservation and variation in the D-Loop-containing region of vertebrate mitochondrial DNA**. *J Mol Biol* 1986, **192**(3):503-511.

24. Quinn TW, Wilson AC: **Sequence evolution in and around the mitochondrial control region in birds**. *J Mol Evol* 1993, **37**(4):417-425.
