# Supplementary figures and images for: Improved tRNA prediction in the American house dust mite reveals widespread occurrence of extremely short minimal tRNAs in acariform mites
Source: BMC Genomics. 2009 Dec 11;10:598. doi: 10.1186/1471-2164-10-598 (PMC2797822; doi:10.1186/1471-2164-10-598)

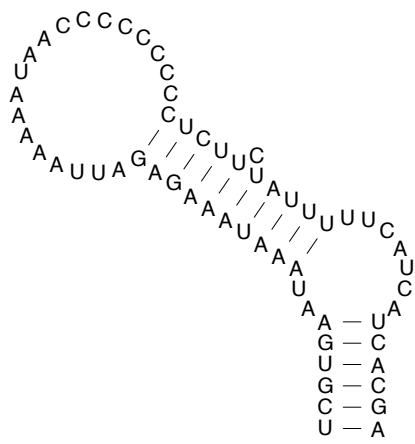

*D. farinae*

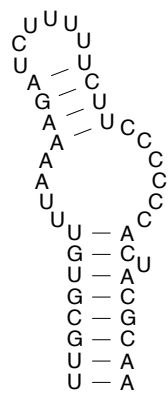

*D. pteronyssinus*

Supplement: Additional file 4 — Secondary structures forming between genes ND6 and ND1 in Dermatophagoides spp. Stem loop structures present between ND6 and ND1 genes in Dermatophagoides farinae and D. pteronyssinus. [file 1471-2164-10-598-S4.PDF]
