# Supplementary material for: Improved tRNA prediction in the American house dust mite reveals widespread occurrence of extremely short minimal tRNAs in acariform mites
Source: BMC Genomics. 2009 Dec 11;10:598. doi: 10.1186/1471-2164-10-598 (PMC2797822; doi:10.1186/1471-2164-10-598)
Supplement: Additional file 6 — Transfer RNAs and minimum free energy structures in Dermatophagoides spp. and Steganacarus magnus. Transfer RNAs of D. farinae (DF), D. pteronyssinus (DP), and S. magnus (SM) found by tRNAscan-SE and ARWEN and alternative free energy structures inferred by mfold. Putative compensatory mutations are indicated by an asterisk. [file 1471-2164-10-598-S6.PDF]

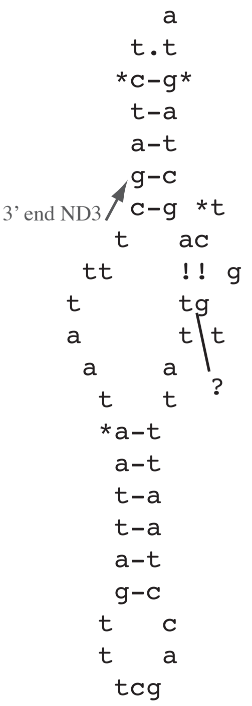

1. Arg-DP_D-loop.
(ARWEN)

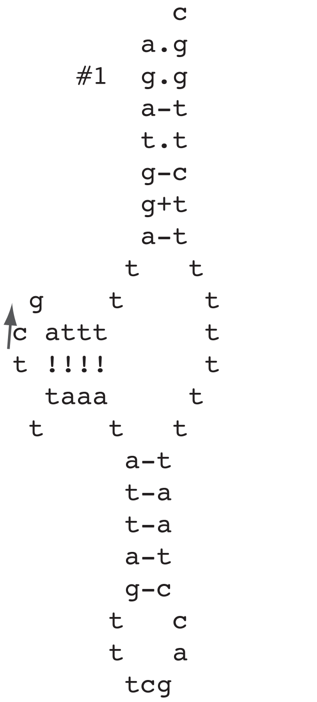

2. Arg-DF_TV-loop.
(alternative 5′ acceptor stem and D-arm)(ARWEN)

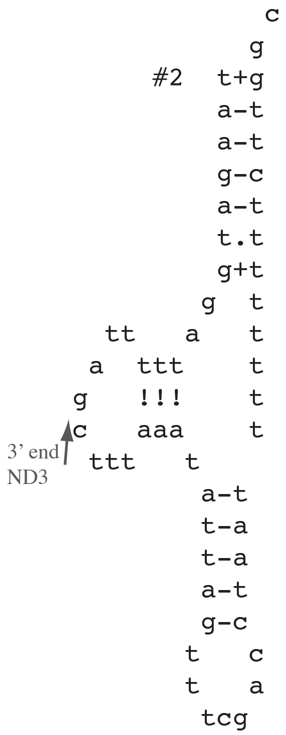

3. Arg-DF_TV-loop.
(alternative 5′ acceptor stem and D-arm)(ARWEN)

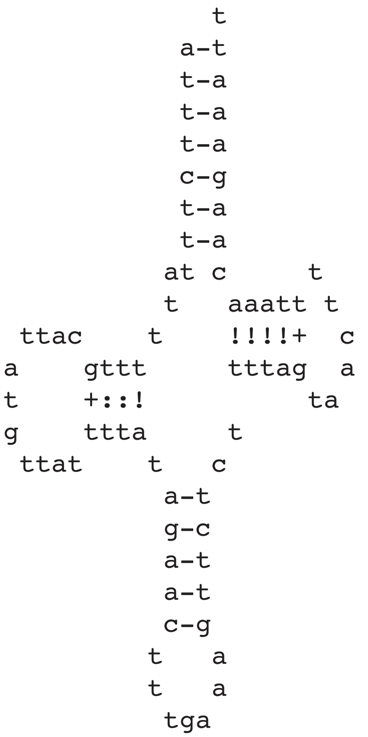

4. Ser2-SM_cloverleaf
(ARWEN)

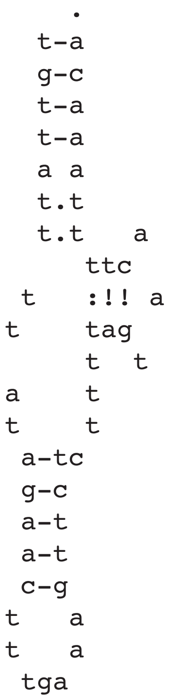

5. Ser2-SM_D-loop
(ARWEN, preferred)

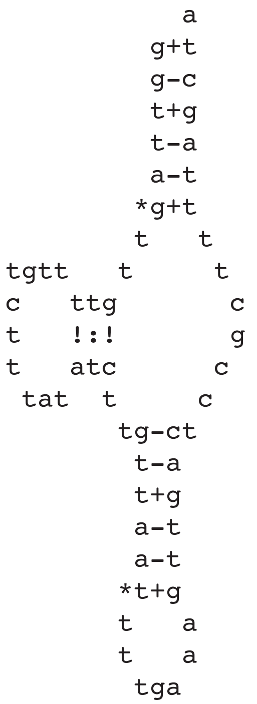

6. Ser2-DF_TV_loop.
(ARWEN, alternative)

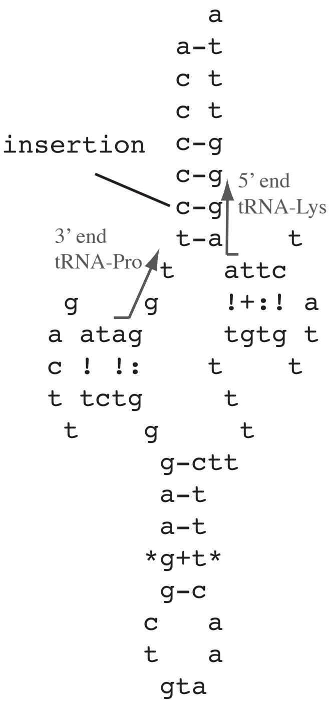

7. Tyr-DF_clover. search 1 (ARWEN)

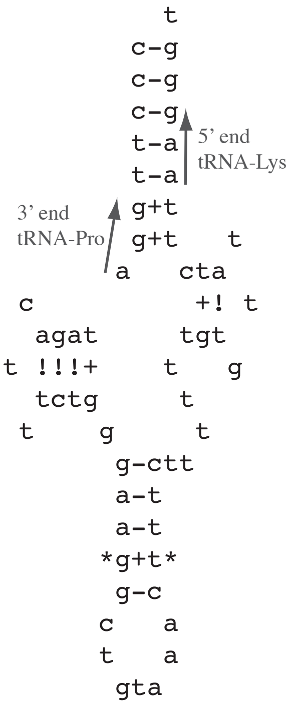

8. Tyr-DF_clover. search 3 (ARWEN)

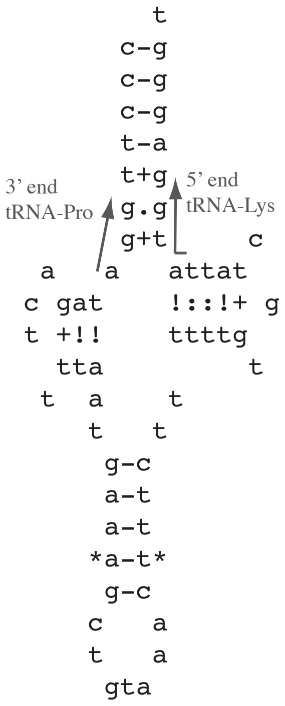

9. Tyr-DP_clover. search 3 (ARWEN)

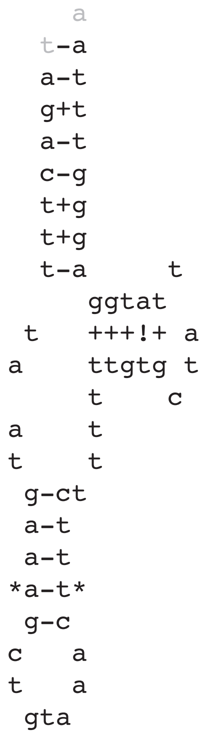

10. Tyr_DP_D-loop. search 2 (ARWEN)

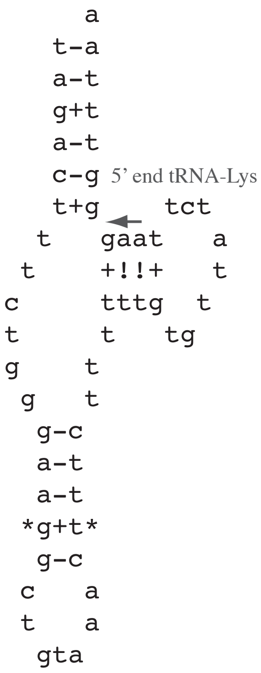

11. Tyr_DF-D-loop. search 1 (ARWEN)

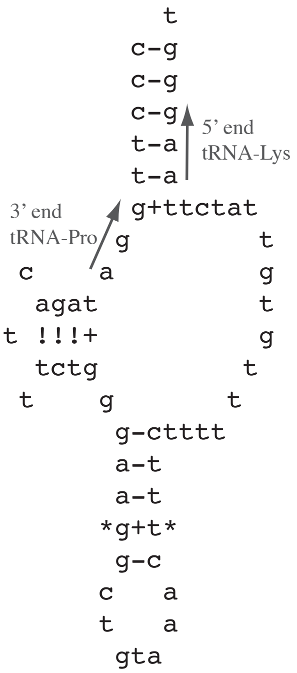

12. Tyr-DF_TV-loop. search 3 (ARWEN)

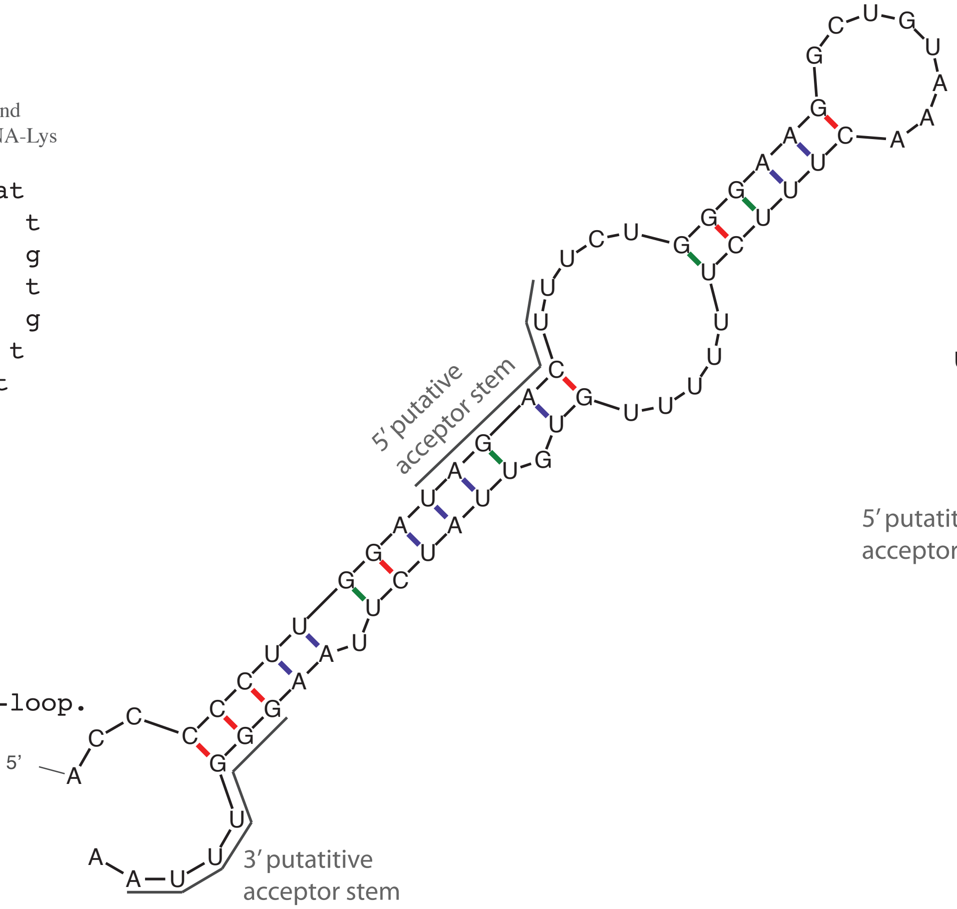

13. DF_mfold

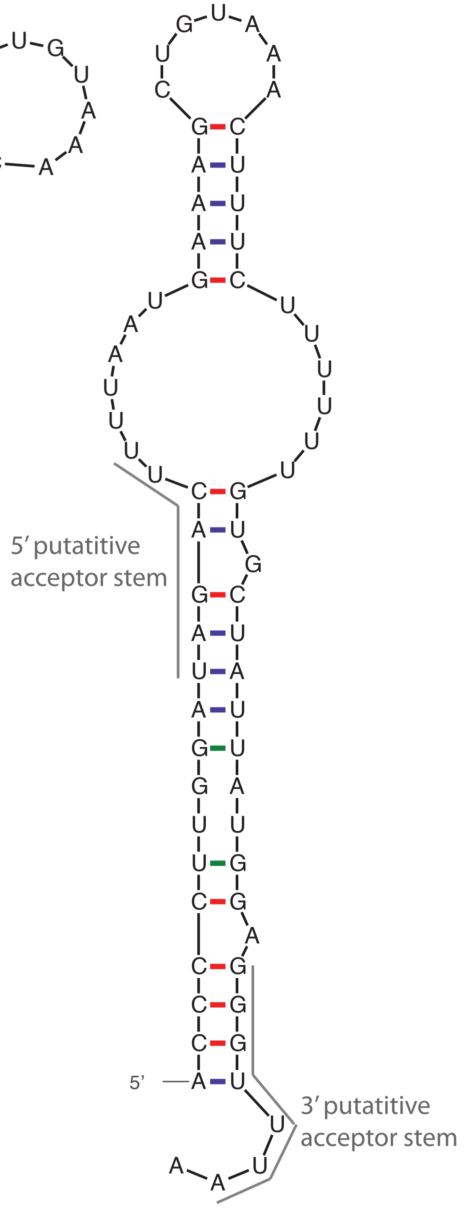

14. DP_mfold

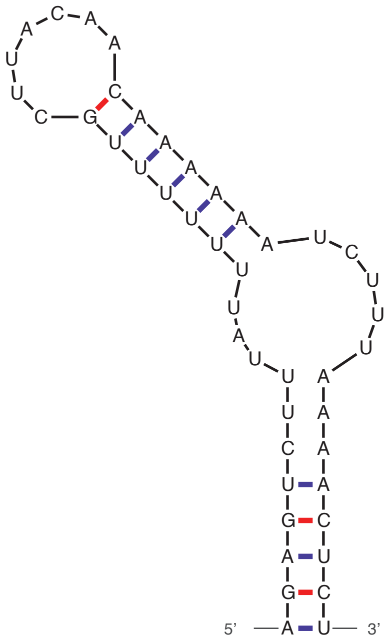

15. tRNA-Val_DF (mfold)

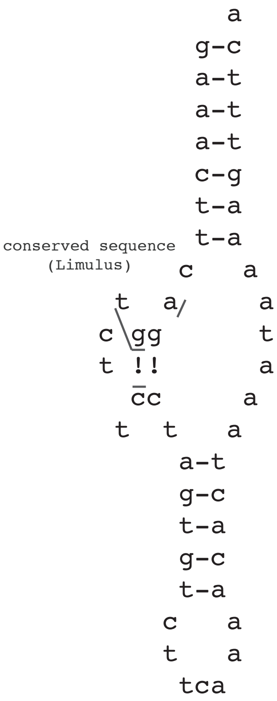

16. Trp_SM (ARWEN)

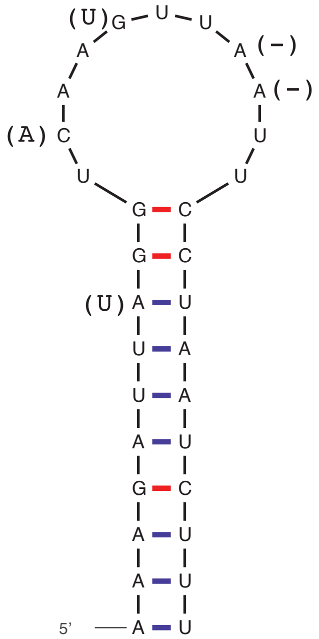

17. Trp-ND1 stem-loop_DF (DP in parentheses)

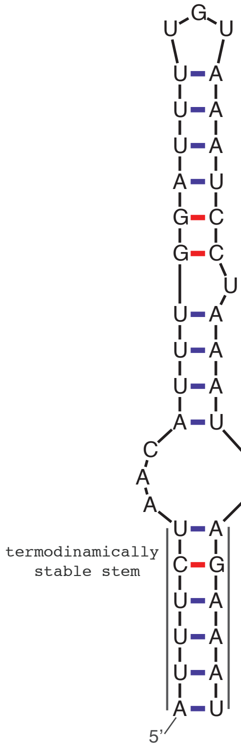

18. Thr (ND4L-6 structure)_SM (mfold)

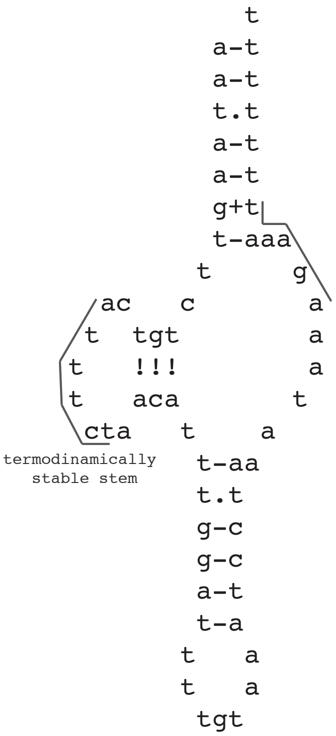

19. Thr_TV-loop_SM (ARWEN)

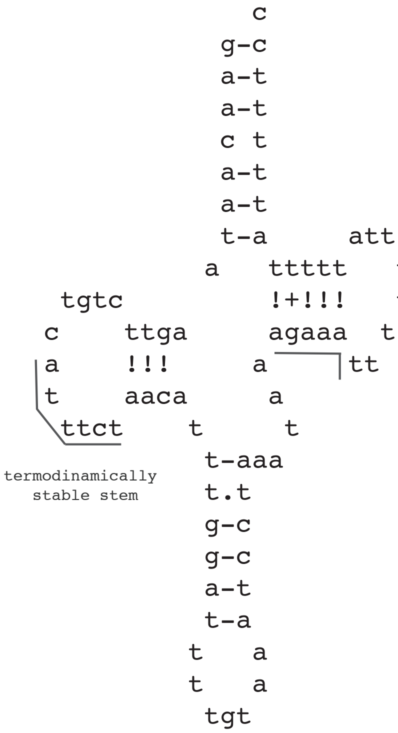

20. Thr_cloverleaf_SM (ARWEN)

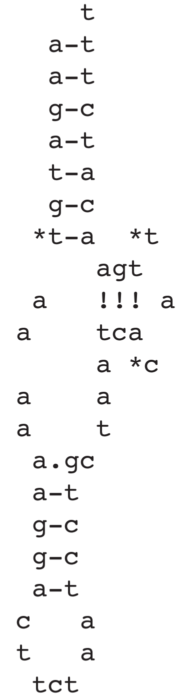

21. Ser1_DP, dangling c not shown (ARWEN)
