# Supplementary material for: Improved tRNA prediction in the American house dust mite reveals widespread occurrence of extremely short minimal tRNAs in acariform mites
Source: BMC Genomics. 2009 Dec 11;10:598. doi: 10.1186/1471-2164-10-598 (PMC2797822; doi:10.1186/1471-2164-10-598)
Supplement: Additional file 8 — Alignment of the region between genes ND6 and ND4L for Dermatophagoides spp. and Steganacarus magnus. Secondary structure information of tRNA-Thr is indicated by parenthesis, anticodons are color-coded (blue Dermatophagoides spp.; red S. magnus). The alignment suggests that the original tRNA-Pro of S. magnus was probably inferred incorrectly. [file 1471-2164-10-598-S8.DOC]

**Alignment of the region between ND6 and ND4L**

ND6<(((((((--------(((((-------())))-(((---------)))--)))))))------>ND4L

CAA<TCCTCTTAA-TGCTTCTGATTTACAAAATCAGGGTTATCT--TAGAACTTAAGAGGAGTTTAA>ATG

CAA<TCCTTAAAAATTCTTTTGATTTACAAAATCAATGTTCTTTATTTAAACTACAAAGGA---AAA>ATG

-------ATTTCTTTATTTAGGATTTACAAAATCCAAATGTTAGAAAT----------------------

Legend:

ND6<=start of ND6 (complement)

>ND4L=start of ND4L

ACA=anticodon of tRNA-Thr (complement), *Dermatophagoides farinae* and *D. pteronyssinus*, respectively

() stem regions of tRNA-Thr (complement)

ATTTCT = putative stems of *Steganacarus magnus*

AGG = anticodon of putative tRNA-Pro (as originally inferred) of *Steganacarus magnus*

Starts of ND6 and ND4L of *Steganacarus magnus* not shown because they can not be aligned with *Dermatophagoides*.
