# Supplementary material for: Improved tRNA prediction in the American house dust mite reveals widespread occurrence of extremely short minimal tRNAs in acariform mites
Source: BMC Genomics. 2009 Dec 11;10:598. doi: 10.1186/1471-2164-10-598 (PMC2797822; doi:10.1186/1471-2164-10-598)
Supplement: Additional file 9 — General oligonucleotide primers used to amplify four gene fragments of Dermatophagoides farinae mitochondrial genome. Primer sequences are written from the 5' end. [file 1471-2164-10-598-S9.DOC]

## Table S1 - General oligonucleotide primers used to amplify four fragments of Dermatophagoides farinae mitochondrial genome

| **Primer Name[[1]](#endnote-2)** | **5'-M13 tail[[2]](#endnote-3)** | **Primer sequence (no M13 tail)** | **PCR[[3]](#endnote-4)** |
| --- | --- | --- | --- |
| COX1_16F |  | TGANTWTTTTCHACWAAYCAYAA | CO1.p (F) |
| COX1_1324R |  | CDGWRTAHCGDCGDGGTAT | CO1.p (R) |
| COX1_25Fshort_T | M13FORW | TCHACWAAYCAYAARRAYA | CO1.n (F) |
| COX1_1282R_T | M13REV | CCWVYTARDCCTARRAARTGTTG | CO1.n (R) |
| COX1_220F |  | ATRATHGGNGGNTTYGGIAA | CO1-2.p (F) |
| CO2_581R |  | CCRCARATYTCTGARCAYTGWC | CO1-2.p,h (R) |
| COX1_705F_T | M13FORW | TTGATTTTTTGGTCATCCAGA | CO1-2.h (F)[[4]](#endnote-5) |
| 12S_ACRF_125F |  | TCRRATNKYGTGCCAGCAGT | 12S-16S.p (F)[[5]](#endnote-6) |
| 16S_ACRF_1155R |  | TWWWWAGATAARAWCCARTCTG | 12S-16S.p (R)^e^ |
| 12S_ACRF_134F |  | GTGCCAGCAGTYKCGGTYAT | 12S-16S.sh (F)^e^ |
| 16S_ACRF_1132R_T | M13FORW | YRCCGRYYTGAACTCAGH | 12S-16S.sh (R)^e^ |
| ND4_626F |  | YTWCCWAARGCHCATGTDGA | ND4-ND5.p,h (F) |
| ND5_675R |  | TNACHARDGTDGAAGAATGRAC | ND4-ND5.p (R) |
| ND5_600R |  | AADGGAADYTGDGCICTYTT | ND4-ND5.h (R) |
| CytB_412F |  | AGGNTAYGTTYTTCCITGRG | COB.p (F) |
| CytB_847R |  | GGRATAGAWCGWARRATDGC | COB.p (R) |
| CytB_419F |  | GTTYTTCCTTGRGGICAAAT | COB.sh (F) |
| CytB_818R |  | GCRAATARRAARTATCATTCWGGTT | COB.sh (R) |

1. Primers were designed using alignment of parasitiform and acariform taxa; they are expected to work in these groups, but were tested only in acariform mites [↑](#endnote-ref-2)
2. M13FORW=TGTAAAACGACGGCCAGT, M13REV = CAGGAAACAGCTATGACC; M13 tails were not consistently added to all nested primers; primers with M13 tails were used in other experiments [↑](#endnote-ref-3)
3. product of parent PCR (p) is used for subsequent nested (n), heminested (h), or subheminested (sh) PCRs; (F) = forward primer, (R) = reverse primer [↑](#endnote-ref-4)
4. works but may be optimized further [↑](#endnote-ref-5)
5. specific to Acariformes [↑](#endnote-ref-6)
